# Interactions of separately conserved α-(1→6) glucosidases that participate in maize endosperm starch biosynthesis

**DOI:** 10.1093/plphys/kiaf417

**Published:** 2025-09-23

**Authors:** Susan K Boehlein, Tracie A Hennen-Bierwagen, Stacie L Shuler, William F Tracy, L Curtis Hannah, Marcio F R Resende, Alan M Myers

**Affiliations:** Horticultural Sciences Department, University of Florida, Gainesville, FL 32601, USA; Roy J. Carver Department of Biochemistry, Biophysics, and Molecular Biology, Iowa State University, Ames, IA 50011, USA; Department of Agronomy, College of Agricultural and Life Sciences, University of Wisconsin, Madison, WI 53076, USA; Department of Agronomy, College of Agricultural and Life Sciences, University of Wisconsin, Madison, WI 53076, USA; Horticultural Sciences Department, University of Florida, Gainesville, FL 32601, USA; Horticultural Sciences Department, University of Florida, Gainesville, FL 32601, USA; Roy J. Carver Department of Biochemistry, Biophysics, and Molecular Biology, Iowa State University, Ames, IA 50011, USA

## Abstract

Chloroplast-containing species possess 2 α-(1→6)-glucosidases that share a common ancestor but were independently acquired by horizontal gene transfer from separate eubacterial donors. The pullulanase-type enzyme (CAZy subfamily GH13_13) and the isoamylase-type enzyme (CAZy subfamily GH13_11) both hydrolyze branch linkages in α-polyglucans. Thus, both enzyme types function as debranching enzymes (DBE) in starch metabolism. As both enzyme types are conserved, distinct selectable functions are expected. This study describes the functional interactions between maize (*Zea mays* L.) pullulanase1 (ZPU1) and the isoamylase-type enzyme complex comprising the paralogous proteins isoamylase1 (ISA1) and isoamylase2 (ISA2). Mutation of ISA1 or ISA2 caused reduced ZPU1 activity in developing endosperm extracts, and the addition of ISA1 to ZPU1-expressing yeast (*Saccharomyces cerevisiae*) cells caused increased ZPU1 activity. Specific amino acid substitutions in ISA1 resulted in altered ZPU1 mobility in SDS-PAGE. In vivo protein–protein interaction tests and co-immunoprecipitation revealed that ZPU1 and ISA1 interact in multi-subunit complexes. Maize lines harboring ISA1 mutations, exhibiting a classical low-starch, high-phytoglycogen-accumulation phenotype, were altered by recurrent selection so that kernel appearance reverted to near normal. Extragenic suppression indicated the requirement for ISA1/ISA2 activity had been bypassed. These results are consistent with a functional overlap between the GH13_11 and GH13_13 DBE types and raise the possibility that multiple GH13 proteins, namely ZPU1, ISA1 and ISA2, act together to physically coordinate their hydrolytic activities on precursor α-polyglucans.

## Teaser

Two conserved classes of plant starch debranching enzyme with distinct prokaryotic origins display regulatory interactions and directly contact each other.

## Introduction

Plant starch not only furnishes the energy for early seed and tuber germination but also is a major contributor to the calorie supply for humans and other animals. Starch is also used for the production of many industrial products including fuel. Despite its importance for life as we know it and its relative simplicity in structure, the fine details of starch biosynthesis are still not fully understood. Chemically, starch is a simple molecule, composed of one type of monomer unit, glucose, joined together in polymers by only 2 different glycoside bonds, specifically α-(1→4) linkages in so-called linear chains and α-(1→6) branch linkages that join linear chains to each other. Starch exists as insoluble granules that contain 2 classes of glucose homopolysaccharides, namely amylose, which is predominantly linear, and the branched polymer amylopectin. Amylose typically contains several hundred to several thousand glucose units linked by α-(1→4) glycoside bonds, with rare α-(1→6) branch points. In the maize (*Zea mays* L.) seed, amylose accounts for approximately 25% of the total starch. Amylopectin, in contrast, is more highly branched with 3–5% of the linkages in the α-(1→6) configuration, and contains approximately 10^5^–10^6^ glucose units. In contrast to glycogen, a soluble glucose homopolymer that occurs in non-plant species, branch points are clustered in amylopectin such that linear chains can form double helices. These assemble into higher order structures that eventually give rise to insoluble starch granules with diameters of 5–10 *μ*m, typical in mature cereal seeds. In maize endosperm, amylopectin accounts for approximately 75% of the mass of starch granules. Amylose and amylopectin are synthesized by parallel pathways, as shown by the finding of Nelson and Rines (1962) that mutations of the maize *waxy* locus (*wx*) eliminate amylose without affecting amylopectin production and its assembly into insoluble granules.

Starch polymers are assembled starting from the glucosyl unit donor ADP-glucose (ADPGlc) by the concerted action of starch synthases (SS; ADP-glucose: 1,4-α-D-glucan 4-α-D-glucosyl transferase) and starch branching enzymes (SBE; 1,4-α-D-glucan: 1,4-α-D-glucan 6-glucosyl transferase). An unexpected observation, which is the subject of this report, is that enzymes that hydrolyze branch linkages, i.e. starch debranching enzymes (DBE; α-D-glucan 1,6-α-D-glucohydrolase), are also required for the accumulation of normal starch levels. Two classes of DBE exist in plants (for reviews, see Nakamura 1996; Manners 1997; Hennen-Bierwagen et al. 2012). These are referred to as isoamylase-type DBE (ISA) or pullulanase-type DBE (PUL) (also known as limit dextrinase), based on similarity to characterized enzymes from eubacteria. Structural similarity and limited primary sequence homology identify PUL and ISA as members of the α-amylase superfamily (Kuriki and Imanaka 1999; MacGregor et al. 2001), designated as family GH13 in the classification system of the Carbohydrate-Active enZyme database (CAZy) (Stam et al. 2006; Drula et al. 2022). The widespread GH13 enzymes are defined by a common catalytic scaffold with a (β/α)_8_-barrel structure that organizes 3 conserved residues to confer a shared double-displacement enzymatic mechanism. Diversity among GH13 subfamilies results in distinctions in substrate specificity and the result of the reaction, i.e. either hydrolysis or transglucosylation. Thus, although ISA and PUL are structurally related and both specifically hydrolyze α-(1→6) linkages, they have acquired different activities regarding the structure of the polymers they act upon. Plant PUL enzymes are members of subfamily GH13_13, and like PUL from eubacterial sources are active in vitro toward the linear substrate pullulan in which maltotriose units are linked to each other by α-(1→6) glycoside bonds i.e. (-Glc_α1,4_-Glc_α1,4_-Glc_α1,6_-)_n_ (Beatty et al. 1999). ISA proteins constitute subfamily GH13_11, and in contrast to PUL are not active with pullulan (Rahman et al. 1998; Kobayashi et al. 2016). ISA and PUL are both active with amylopectin and its α--limit dextrin derivatives, with varying degrees of efficiency. The precise in vivo substrates of ISA or PUL are not known, and it should be noted that although pullulan is useful as an in vitro substrate, it is not native to plants.

Insight regarding DBE function in starch biosynthesis came originally from mutations at the maize *sugary1* locus (*su1*). A *su1-* mutant allele was used by Correns (1901) at the start of the 20th century to help clarify Mendel's rules of inheritance and also was exploited at that time and earlier to form the basis of conventional sweet corn (Tracy et al. 2006). Mutations of the *su1* locus cause elevated monosaccharide and sucrose levels in developing kernels and, uniquely, condition high levels of soluble α-polyglucan, termed phytoglycogen, in which branch linkages are approximately twice as frequent as they are in amylopectin (for review, see Hennen-Bierwagen et al. 2012). Total α-polyglucan in *su1-* mutants is only slightly reduced compared to normal kernels, but granular starch content is strongly diminished so that phytoglycogen accounts for up to 75% of the total α-polyglucan.

Pan and Nelson (1984) found that PUL activity in developing maize endosperm was reduced by ∼75% in *su1-* mutants. This pointed to the surprising inference that cleavage of glycoside bonds is necessary to synthesize wild-type levels of starch. Of the 3 separate PUL activity peaks detected in non-mutant endosperm by hydroxyapatite column chromatography, one was absent from *su1-* mutant tissue, while the other 2 were substantially reduced. All 3 activity peaks are likely provided by the same protein, referred to here as *Zea mays*  pullulanase1 (ZPU1), because only a single gene encoding a pullulanase-type DBE is present in the maize genome and because plant ISA proteins are essentially inactive toward pullulan as an in vitro substrate (Beatty et al. 1999).

The effect of *su1-* mutations on ZPU1 activity was later shown to be indirect. Molecular characterization of the *su1* locus revealed that it does not encode ZPU1 but rather codes for a protein homologous to bacterial ISAs, referred to as isoamylase1 (ISA1) (James et al. 1995). Hence, *Su1* encodes an ISA enzyme, and mutation of this protein results in reduced ZPU1 activity by an unknown mechanism. This effect is post-translational because ZPU1 mRNA and protein are present at near-normal levels in a *su1-* mutant (Beatty et al. 1999).

A series of reports in plant species besides maize, including rice (*Oryza sativa* L.) (Nakamura 1996), potato (*Solanum tuberosum*) (Bustos et al. 2004), barley (*Hordeum vulgare*) (Burton et al. 2002), *Arabidopsis thaliana* (Zeeman et al. 1998), and the evolutionarily distant, unicellular green alga *Chlamydomonas reinhardtii* (Mouille et al. 1996), showed that loss of ISA activity leads to decreased starch content and appearance of phytoglycogen. Thus, the ISA function appears to be a fundamental aspect of starch biosynthesis conserved in all chloroplast-containing species. The relationship between ISA and PUL is also conserved, at least in cereals, because rice mutations in the gene encoding ISA1 caused reduced PUL activity (Nakamura 1996), just as was observed in maize.

Phylogenetic analysis indicates that the genes encoding ISA and PUL were independently incorporated into the genome of a common ancestor of the chloroplast-containing species, i.e. the Chloroplastida, by horizontal gene transfer from separate eubacterial donor species (Deschamps et al. 2008). Conservation of both enzymes in apparently all green algae and land plants implies that their distinct catalytic specificities each provide a selected function. Conservation applies to 3 ISA paralogs, 2 of which, namely ISA1 and isoamylase2 (ISA2), function in starch biosynthesis (for review see Hennen-Bierwagen et al. 2012), and a third, isoamylase3 (ISA3), which is required for starch catabolism in leaves during the night (Wattebled et al. 2005; Delatte et al. 2006). Genetic analyses of PUL revealed both catabolic and anabolic functions. Mutations affecting PUL did not affect starch levels in Arabidopsis leaves (Wattebled et al. 2005; Delatte et al. 2006); however, in maize leaves such mutations conditioned a starch excess phenotype at the end of the dark phase (Dinges et al. 2003a). Double mutants affecting both ISA3 and PUL in Arabidopsis exhibited a strong starch excess phenotype beyond the level of ISA3 mutants alone (Wattebled et al. 2008). Thus, PUL appears to function in starch degradation in a way that overlaps with ISA3.

A biosynthetic role of PUL was also detected, again overlapping with ISA function. Combining PUL mutations with ISA2 defects in Arabidopsis leaf enhanced the degree of the starch decrease/phytoglycogen-accumulation phenotype (Wattebled et al. 2005). Similarly, in maize or rice genetic backgrounds in which ISA1 is partially compromised, PUL mutation enhances this characteristic phenotype (Dinges et al. 2003a, 2003b; Fujita et al. 2009). A biosynthetic role is consistent with the observation that mRNA encoding ZPU1 is most abundant in endosperm during periods of rapid starch accumulation (www.maizegdb.org, gene model Zm00001eb088740). These genetic data indicate PUL is active both in starch biosynthesis and in starch degradation, in each instance overlapping with ISA function(s).

In summary, 2 structurally related classes of DBE with independent evolutionary origins are both strictly conserved in plants and green algae. Each of them contributes to starch accumulation and determination of α-polyglucan structure. In cereal endosperm, the 2 enzymes interact in vivo such that PUL activity is influenced by the state of ISA. The molecular mechanisms responsible for these physiological properties are not known. The current study further characterized the functional relationship of ISA and ZPU1 in maize endosperm. ZPU1 activity was affected in an allele-specific manner by mutations of ISA1 and also by deletion of ISA2, and the state of the ZPU1 protein as detected by mobility in SDS-PAGE was also affected by these mutations in an allele-specific fashion. Direct binding of ISA1 and ZPU1 was demonstrated, and ISA1 was shown to be a positive regulator of ZPU1 enzymatic activity using a heterologous in vivo reconstitution system. Furthermore, maize lines containing yet unidentified second-site suppressor mutations restore ZPU1 activity in *su1-*mutant backgrounds, coincident with restoration of near-normal starch accumulation. The results support functional cooperation of the PUL and ISA classes of DBE in starch biosynthesis, potentially through assembly into heteromeric complexes.

## Results

### ZPU1 activity in endosperm with mutations affecting ISA1/ISA2 heteromeric complexes

Previous analyses found the pullulan hydrolysis rate during incubation with immature maize endosperm extracts is reduced 2- to 3-fold in multiple *su1-* mutants compared to congenic non-mutant controls (Pan and Nelson 1984; Shuler et al. 2017). This activity is presumed to be provided by ZPU1, because the structure of pullulan renders its α-(1→4) bonds impervious to any known α- or β-amylase, and its α-(1→6) bonds are not substrates of recombinant ISA1 (Rahman et al. 1998). Here, the same analysis was extended to additional *su1* alleles with known effects on ISA1, and to lines lacking ISA2 or ZPU1 (Table 1). Mutants and congenic standards were compared in multiple inbred genetic backgrounds. The assays utilized Red Pullulan, in which pullulan is covalently modified by attachment of a dye, such that hydrolysis of α-(1→6) bonds releases modified maltotriose that is quantified after removing the remaining polymer. In all instances, the product concentration was linear with time, and the hydrolysis rate was proportional to extract volume, indicating ZPU1 activity was rate-limiting.

**Table 1.** Alleles of *su1*, *isa2*, and *zpu1* used in this study

Locus^a^	Encoded protein	Allele name	Allele structure^a^	Effect	Inbred(s)^b^	References^c^
*su1*	ISA1	*Su1*	Non-mutant allele	Non-mutant protein	W64A, Ia453, P39, A632	(1)
		*su1-Ref*, (*su1-NE*)	T to C transition at nt 6830	Replaces Trp 578 by Arg (W578R)	W64A, Ia453, P39, A632, Wpse2, Wpse3	(2)
		*su1-st*	Transposon after nt 3509, aberrant pre-mRNA splicing	Low protein level, partially functional	W64A	(3)
		*su1-am*	G to T transition at nt 2541	Replaces Arg 308 by Ile (R308I)	W64A	(4)
		*su1-Bn2*	C to G transversion at nt 7626	Replaces Asn 628 by Lys (N628K)	W64A	(5)
		*su1-NC*	C to T transition at nt 6171	Replaces Arg 504 by Cys (R504C)	A632	(6)
		*su1-SW*	A to G transition at nt 6780	Replaces Asn 561 with Ser (N561S)	A632	(6)
		*su1-4582*	Transposon after nt 504	Null, no protein product	W64A	(7)
*isa2*	ISA2	*Isa2*	Non-mutant allele	Non-mutant protein	W64A, Ia453	(8)
		*isa2-339*	Transposon after nt 1050	Null, no protein product	W64A, Ia453	(4)
*zpu1*	ZPU1	*Zpu1*	Non-mutant allele	Non-mutant protein	W64A	(9)
		*zpu1-204*	Transposon after nt 368	Null, no protein product	W64A	(10)

^a^Gene loci refer to inbred B73 in the Zm–B73–REFERENCE-NAM–5.0 genome sequence (www.maizegeb.org). The *su1* locus corresponds to gene model Zm00001eb174590. The *isa2* locus corresponds to gene model Zm00001eb287400, and the *zpu1* locus corresponds to gene model Zm00001eb088740. Nucleotide and amino acid numbers refer to canonical transcripts from the indicated gene models.

^b^Listed inbreds are those into which the indicated allele has been introgressed by repeated backcrossing.

^c^References to allele structure are as follows:

(1) Gene model Zm00001eb174590 in the Zm-B73-REFERENCE-NAM-5.0 genome sequence (www.maizegdb.org);

(2) Correns, 1901; Dinges et al., 2001; Whitt et al., 2002; Tracy et al., 2006; De Vries et al., 2016;

(3) Dinges et al., 2001;

(4) Kubo et al., 2010;

(5) Hennen-Bierwagen et al., 2025;

(6) Tracy et al., 2006;

(7) James et al., 1995;

(8) Gene model Zm00001eb287400 in the Zm-B73-REFERENCE-NAM-5.0 genome sequence (www.maizegdb.org);

(9) Gene model Zm00001eb088740 in the Zm-B73-REFERENCE-NAM-5.0 genome sequence (www.maizegdb.org);

(10) Dinges et al., 2003a.

**Figure 1.**
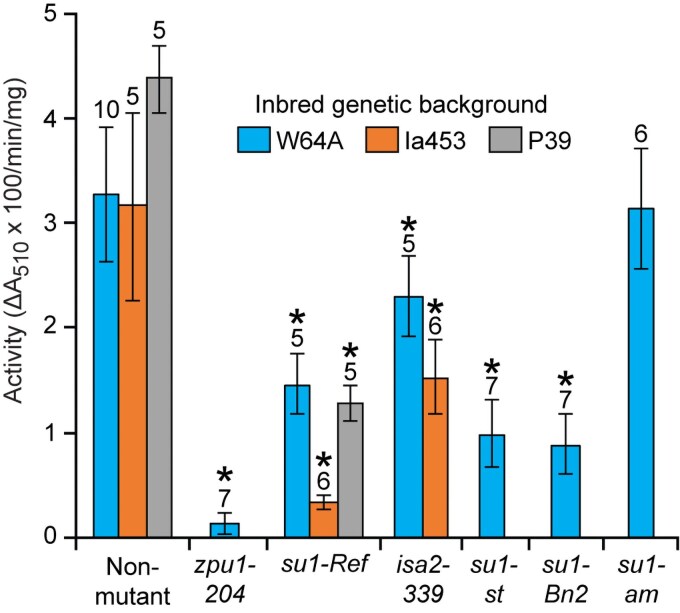
ZPU1 Activity in total soluble endosperm extracts. Mutant lines and congenic non-mutants are compared in 3 different inbred backgrounds, as indicated. Activity units indicate hydrolysis of alpha-1,6-glycoside bonds in Red Pullulan. Values are average ± standard deviation from the indicated number of biological replicates of individual kernels from a single homozygous ear of each genotype. Asterisks (*) indicate significant difference from the congenic non-mutant standard (Student's *t* test, *P* value < 0.003).

ZPU1 activity was measured in extracts of immature endosperm tissue (Fig. 1) from kernels harvested 20 days after pollination (DAP) that had been stored at -80 ℃. Endosperm homozygous for the null allele *zpu1-204* exhibited approximately 5% of the apparent Red Pullulan hydrolysis rate of the congenic non-mutant, indicating the background level of the assay and confirming that ZPU1 provides the great majority of activity detected. The allele *su1-Ref* conditioned approximately 60% reduction of ZPU1 activity in 2 inbred backgrounds, consistent with previously published results (Pan and Nelson 1984; Shuler et al. 2017), and approximately 90% reduction in the Ia453 inbred background that is derived from sweet corn varieties (De Vries and Tracy 2016). The alleles *su1-st* and *su1-Bn2* conditioned reduced ZPU1 activity to approximately the same extent as *su1-Ref*, whereas *su1-am* did not have this effect. The *su1-am* allele, first described by Mangelsdorf (1947), differs from the other alleles tested because it conditions a near-normal endosperm starch phenotype, whereas the other mutants accumulate substantial levels of phytoglycogen.

Mutations affecting ISA2 might also affect ZPU1 activity, because ISA2 assembles into heteromeric complexes that also contain ISA1 (Kubo et al. 2010; Utsumi et al. 2011; Trimble et al. 2016). To test for such an effect, the null allele *isa2-339* was introgressed into the W64A or Ia453 standard background, both of which are homozygous for a non-mutant allele at the *su1* locus. In W64A, deletion of ISA2 had a moderate but significant effect of approximately 25% reduction in ZPU1 activity (Fig. 1). In the Ia453 background, *isa2-339* resulted in approximately 50% reduction in this activity. Thus, changing function of the ISA1/ISA2 heteromultimeric complex by either point mutation within ISA1 or deletion of ISA2 results in decreased ZPU1 activity in total endosperm extracts. This effect of ISA2 deletion occurs even though the active ISA1 homodimer remains in *isa2-339* mutants (Kubo et al. 2010).

### ISA1 mutations affect ZPU1 electrophoretic mobility

**Figure 2.**
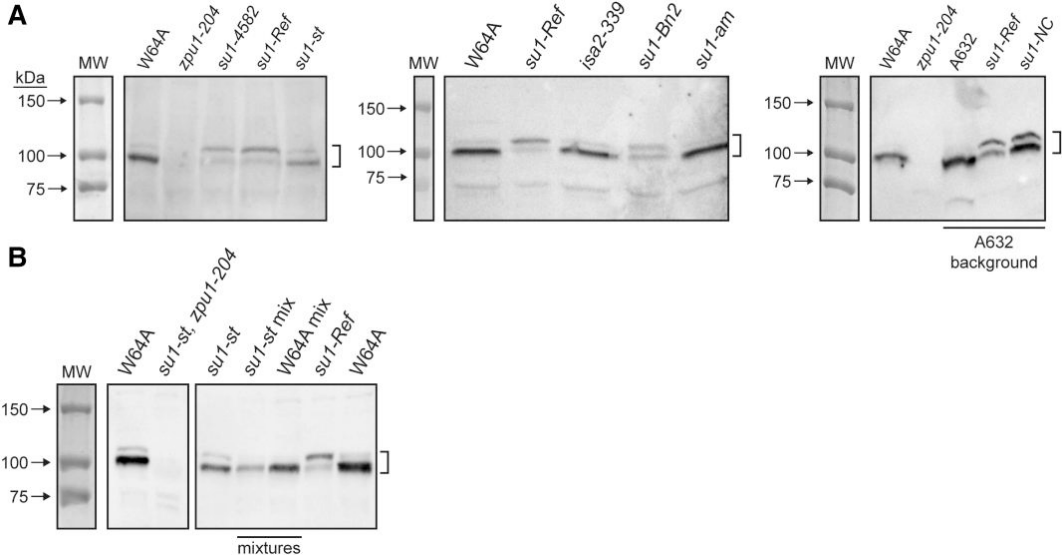
Immunoblot analysis. Total soluble endosperm extracts (30 *µ*g) from kernels harvested 20 DAP were fractionated by SDS-PAGE and probed with anti-ZPU1 antiserum. “MW” indicates stained molecular weight standards on the immunoblot filter. Brackets indicate the positions of ZPU1 bands. **A)** ZPU1 mobility. Maize lines were congenic in the W64A inbred background, except for 3 lines in the A632 inbred background, as indicated. Coomassie blue–stained duplicate gels, indicating consistent protein loads, are shown in Supplementary Fig. S1. **B)** Mixing experiment. Extract from a *su1-st, zpu1-204* double mutant was mixed 1:1 with that from a *su1-st* single mutant or W64A non-mutant standard. *su1-Ref* extract was included as a control.

Immunoblot analysis tested whether mutations affecting ISA1 or ISA2 condition any change in the state of the ZPU1 polypeptide. Total soluble endosperm extracts fractionated by SDS-PAGE were probed with antiserum raised against recombinant ZPU1 (Beatty et al. 1999). Specificity of the anti-ZPU1 serum was confirmed by detection in non-mutant endosperm extracts of a protein of approximately 100 kDa, in agreement with the predicted molecular mass of ZPU1, and the absence of that signal from endosperm homozygous for the null mutation *zpu1-204* (Fig. 2A). Non-mutant inbreds also reproducibly exhibit a far less abundant, minor form of ZPU1 that migrates in SDS-PAGE slightly slower than the major form (Fig. 2). The minor band is also absent from *zpu1-204* endosperm, confirming it is a variant form of ZPU1 and not an unrelated protein that cross-reacts with anti-ZPU1 serum. Four independent *su1-* mutations resulted in obvious changes in the relative intensity of the 2 bands, such that the slower-migrating form increases in abundance while the faster-migrating form decreases (Fig. 2). Alleles that cause this effect are the null mutation *su1-4582* and the missense mutations *su1-Ref*, *su1-Bn2*, and *su1-NC* (Table 1). The effect of *su1-Ref* was observed in 2 inbred backgrounds. The relative abundance of the 2 forms of ZPU1 was not altered in *su1-st* or *su1-am* mutants, nor in endosperm lacking ISA2. Several of the *su1-* mutant alleles also cause reduced total ZPU1 abundance as judged by immunoblot (Fig. 2) (Supplementary Fig. S1).

Mutant endosperms that exhibited altered ZPU1 electrophoretic mobility also accumulated high levels of phytoglycogen, raising the possibility that the presence of soluble α-polyglucan in the extract is responsible for the effect. This was tested by mixing an extract that contains phytoglycogen but lacks ZPU1 protein with extracts exhibiting normal ZPU1 mobility. Double mutants homozygous for both *su1-st* and *zpu1-204* accumulate phytoglycogen. Equal volumes of this mutant extract were mixed with those from a wild-type strain or a *su1-st* single mutant, both of which display normal ZPU1 electrophoretic mobility. After incubation at room temperature for 30 min, the mixtures were fractionated by SDS-PAGE and probed with anti-ZPU1 serum (Fig. 2B). The results confirmed the absence of ZPU1 in the *su1-st, zpu1-204* double mutant and the normal distribution of the 2 electrophoretic mobility forms in the wild-type or *su1-st* single mutant controls. The addition of the phytoglycogen-containing extract did not affect ZPU1 mobility in either wild type or *su1-st*. Thus, at least in this in vitro context, binding to phytoglycogen does not explain the observed alteration in ZPU1 electrophoretic mobility.

### Physical interactions among ISA1, ISA2, and ZPU1

#### Protein–protein interaction tests in vivo

Two-hybrid analysis in yeast (*Saccharomyces cerevisiae*) tested for direct interactions between ISA1, ISA2, and ZPU1. Synthetic sequences optimized for yeast codon utilization were generated that code for the known or predicted mature proteins (Supplementary Table S1) (Supplementary Fig. S2). ISA1 begins at residue 50 relative to the primary translation product, which is the known mature amino terminus (Rahman 1998). Two amino termini were selected for ISA2 based on computational predictions of the cleavage site, designated as ISA2-S or ISA2-L. ZPU1 was initiated at residue 80 relative to the primary translation product. Each protein was fused to the DNA-binding domain of the *S. cerevisiae* GAL4 transcription factor (BD), or to the GAL4 transcriptional activation domain (AD). Plasmids encoding the fusion proteins were introduced into haploid yeast strains that require reconstituted GAL4 activity for growth in the absence of either adenine or histidine, or in the presence of the antifungal antibiotic Aureobasidin A (AbA).

Haploid strains were mated, and then diploids containing both an AD and BD plasmid were selected by complementing auxotrophies for growth in the absence of leucine and tryptophan (-Leu, -Trp). Equivalent aliquots of diploid cells were plated on a second plate that also lacked histidine (-Leu, -Trp, -His) and thus selected for reconstituted GAL4 to activate a synthetic histidine biosynthetic gene. A third plate required 3 independent synthetic reporter genes to be activated by reconstituted GAL4, simultaneously conferring adenine prototrophy, histidine prototrophy, and AbA resistance (-Leu, -Trp, -His, -Ade, and +AbA). Any combination of ISA1 with itself, with ISA2-S, or with ISA2-L conditioned growth on either the relaxed (-Leu, -Trp, -His) or stringent (-Leu, -Trp, -His, -Ade, and +AbA) test plate (Table 2) (Supplementary Fig. S3A), indicating direct binding of ISA1 to itself and to ISA2. These data are consistent with the demonstrated existence of ISA1 homodimers and ISA1/ISA2 heteromultimers in maize, rice, and Chlamydomonas (Utsumi and Nakamura 2006; Kubo et al. 2010; Utsumi et al. 2011; Sim et al. 2014) and ISA1/ISA2 heteromultimers in Arabidopsis leaf and developing kidney bean seeds (Takashima et al. 2007; Facon et al. 2013; Sundberg et al. 2013).

**Table 2.** In vivo protein–protein interaction test results^a^

GAL4-AD fusion	GAL4-BD fusion			
	ISA1	ISA2-S	ISA2-L	ZPU1
ISA1	**++**	**++**	**++**	**−**
ISA2-S	**++**	**+**	**+**	**−**
ISA2-L	**++**	**+**	**+**	**−**
ZPU1	**++**	**−**	**−**	**−**

^a^“++” indicates growth both on relaxed test plates (-Leu, -Trp, and -His) and stringent test plates (-Leu, -Trp, -His, -Ade, and +AbA plates). “+” indicates growth on relaxed test media but not on stringent test media. “**−**” indicates failure to grow on either test plate.

ISA2 also bound to itself in the context of the in vivo protein–protein interaction test. All 4 possible combinations of ISA2-S(AD), ISA2-S(BD), ISA2-L(AD), and ISA2-L(BD) were tested, thus providing multiple biological replications of the experiment. In every instance, the diploids grew on the relaxed test plate (-Leu, -Trp, and -His) but not on the stringent test plate that requires the activity of all 3 reporter genes (-Leu, -Trp, -His, -Ade, and +AbA) (Table 2) (Supplementary Fig. S3B). The single prototrophy selection is a reliable marker for protein–protein interaction because none of the fusion proteins alone, or in control pairwise combinations, conditioned growth on medium lacking histidine (Table 2) (Supplementary Fig. S3C). Failure of ISA2/ISA2 combinations to simultaneously activate 3 reporter genes, even though it can activate the histidine prototrophy marker in isolation, may be explained by a binding affinity less than that of combinations involving ISA2 and ISA1.

**Figure 3.**
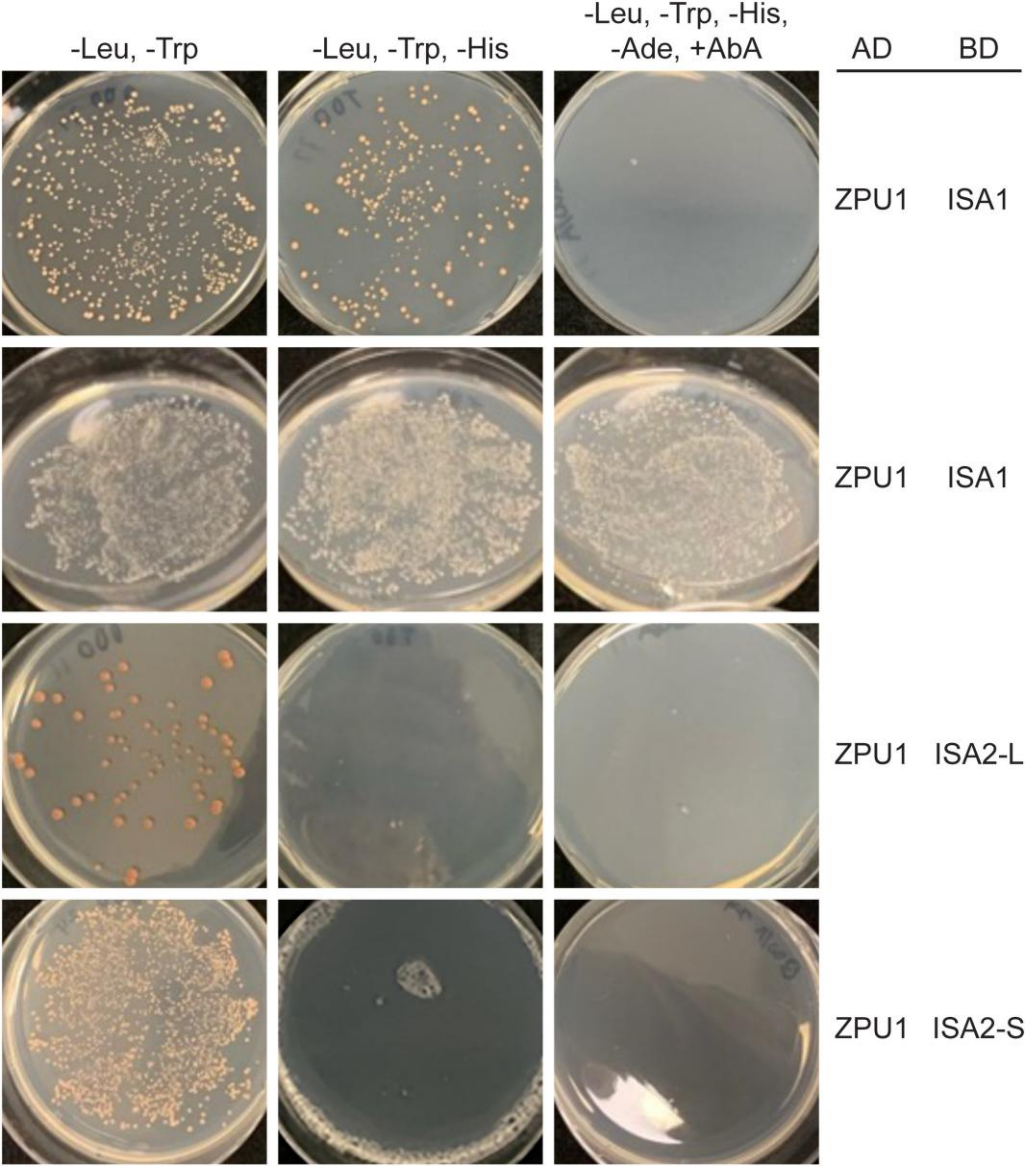
In vivo protein–protein interaction tests. The -Leu, -Trp plate selects for diploids created by mating of haploids expressing GAL4 activation domain (AD) fusions to partners expressing GAL4 binding domain (BD) fusions. Approximately equal numbers of diploid cells were separately deposited on 2 different selective media plates. Colonies were grown for 2 d at 30 ℃ prior to photographing. The top 2 rows are from independent assays beginning with biological replicate transformations. “AbA” indicates Aureobasidin A. Additional in vivo protein–protein interaction test results are shown in Supplementary Fig. S3.

Potential binding interactions between ZPU1 and ISA1 or ISA2 were then tested (Fig. 3). Combination of ZPU1(AD) with ISA1(BD) invariably conditioned growth when only histidine prototrophy was selected and, in some independent repetitions of the assay, simultaneously activated all 3 reporter genes. Combination of ZPU1(AD) with ISA2-S(BD) or ISA2-L(BD) failed to activate any of the reporters. Thus, in this context, ZPU1 binds to ISA1 but not to ISA2.

The results of the in vivo protein–protein interactions are summarized in Table 2, showing: (1) ISA1 binds to itself, ISA2, and ZPU1, (2) ISA2 binds to itself, and (3) ISA2 and ZPU1 do not bind to each other in this context. No other maize proteins are present in the yeast cells, so these binding interactions most likely are direct rather than containing any other protein as an intermediary.

#### Co-immunoprecipitation

**Figure 4.**
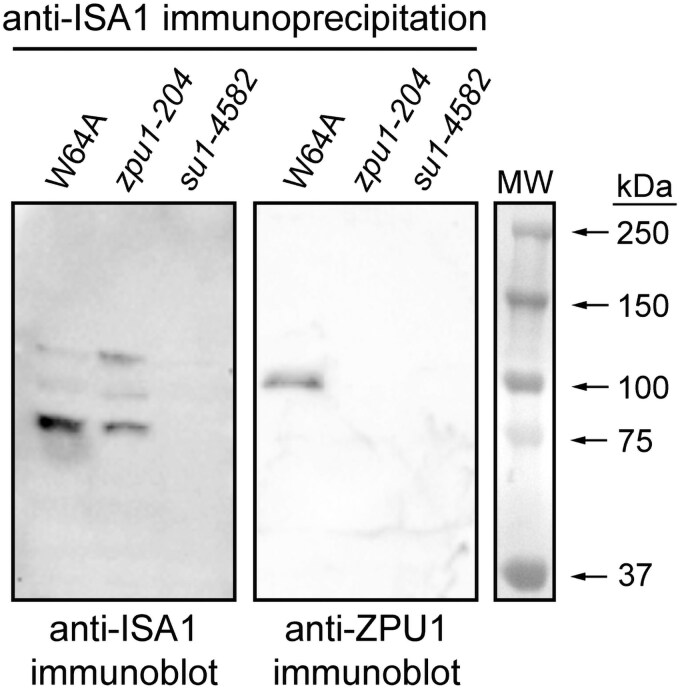
Co-immunoprecipitation. Total soluble endosperm extract from the indicated homozygous mutants and the congenic non-mutant standard were incubated with anti-ISA1 IgG, then proteins bound to the antibodies were precipitated by binding to protein A-Sepharose. Immunoprecipitated proteins were separated by SDS-PAGE and then probed in immunoblots with the indicated antibodies. “MW” indicates pre-stained protein molecular weight standards run in the same gel and transferred to the immunoblot filter.

The binding between native ZPU1 and ISA1 was tested by co-immunoprecipitation from endosperm extracts. Wild-type or mutant extracts were incubated with purified IgG that binds to ISA1. Specificity of the anti-ISA1 IgG fraction was demonstrated by immunoblot analysis that detected a protein corresponding to the known electrophoretic mobility of ISA1 in wild-type endosperm extracts and complete absence of this signal in extracts from endosperm homozygous for the null allele *su1-4582* (Supplementary Fig. S4). Proteins bound to the antibodies were collected by affinity to immobilized protein A. Immunoprecipitated proteins were separated by SDS-PAGE and probed in immunoblots for the presence of ISA1 or ZPU1 (Fig. 4). Anti-ISA1 detected a predominant protein in non-mutant extracts that migrated in SDS-PAGE as expected for ISA1, and 2 less abundant proteins with reduced mobility. All of these signals were absent in the immunoprecipitated fraction from *su1-4582* endosperm that does not contain ISA1, confirming that the antiserum effectively precipitates the expected target. The 3 ISA1 bands were also immunoprecipitated from extracts of *zpu1-204* mutant endosperm that lacks ZPU1.

The same immunoprecipitated fractions were probed with anti-ZPU1 serum. A single protein was detected in the immunoprecipitated fraction from non-mutant extracts that migrated as expected for ZPU1. That protein was absent in the immunoprecipitated fraction from *zpu1-204* mutant endosperm, confirming its identity as ZPU1. The ZPU1 signal depended on the presence of ISA1 in the extracts because it was not detected in the immunoprecipitated fraction from a *su1-4582* mutant. These results indicate that ZPU1 and ISA1 are present together in a complex that is precipitated by anti-ISA1 IgG. Taken together, the in vivo protein–protein interaction test and the co-immunoprecipitation result demonstrate that ISA1 and ZPU1 are present together in a stable complex in the native tissue and bind directly to each other in the reconstituted environment in yeast cells.

The reciprocal experiment, in which the initial immunoprecipitation was with anti-ZPU1 antibodies, was not possible because the crude serum failed to collect ZPU1 from endosperm extracts. The anti-ISA1 antibody, in contrast, is an affinity-purified, highly concentrated IgG fraction, and this may explain why it was effective for immunoprecipitation when the anti-ZPU1 crude serum was not.

### α-(1→6)-Glucosidase activity in a reconstituted in vivo system

Genome engineering of *S. cerevisiae* has been employed previously as a heterologous in vivo system for expression of starch biosynthetic enzymes from plants (Pfister et al. 2016, 2022; Boehlein et al. 2023; Liu et al. 2023). The host genome lacks functional endogenous elements coding for any known α-glucosidase, including glycogen debranching enzyme or maltase (Supplementary Table S2). The host genes encoding glycogen synthase, glycogen branching enzyme, glycogenin, and glycogen phosphorylase are also disrupted. Detection in cell extracts of hydrolytic activity toward α-polyglucans, therefore, is expected to depend on expression of exogenous genes. Synthetic open reading frames coding for maize ISA1 and/or ZPU1 (Supplementary Fig. S2) were attached to a galactose-inducible yeast promoter and a yeast terminator sequence (Supplementary Table S3), and those complete transcription units were integrated into the host genome. Strains were constructed that contain either ISA1 or ZPU1 alone or the 2 maize enzymes together (Supplementary Table S2).

**Figure 5.**
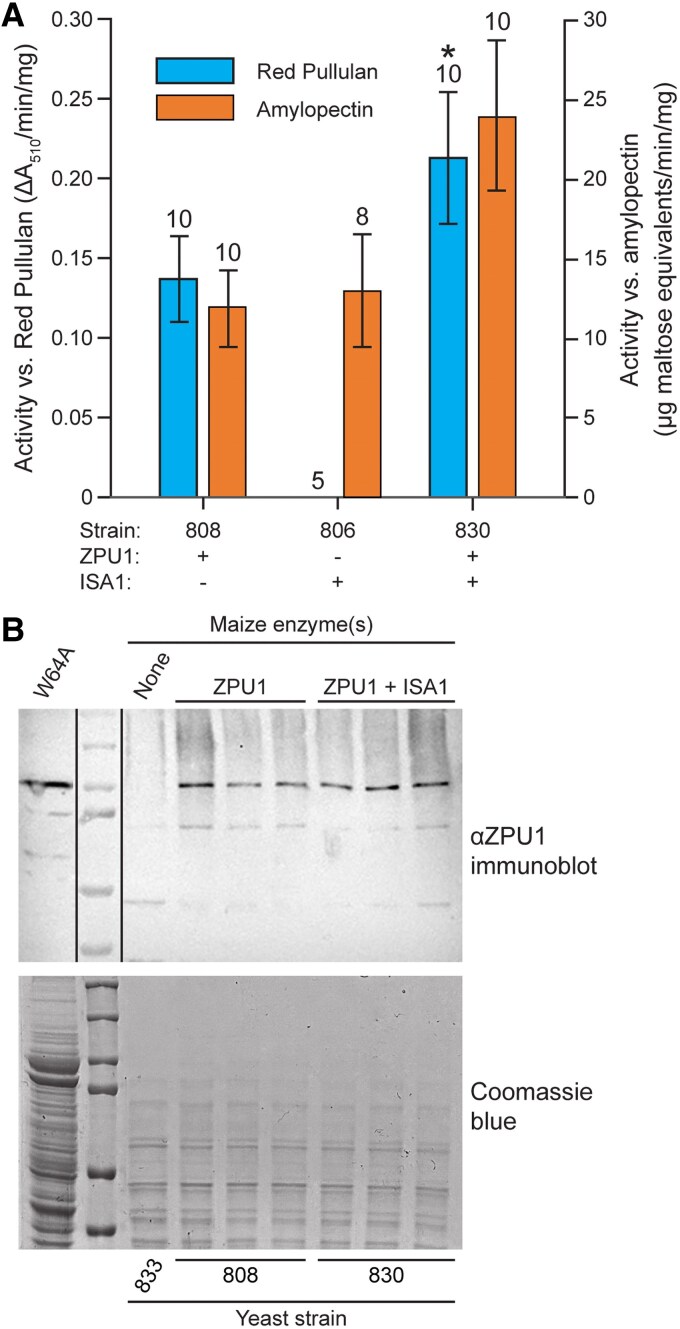
α-(1→6)-Glucosidase activity in total soluble yeast extracts. **A)** Hydrolytic activity measured using Red Pullulan (left axis) or solubilized amylopectin (right axis) as the substrate. Values are average ± standard deviation calculated from the indicated number of replicate assays, each starting from a separate single colony. Yeast strain genotypes are specified in Supplementary Table S2. Strain 806 did not exhibit detectable activity with Red Pullulan. Asterisk (*) indicates significant difference from strain 808 lacking ISA1 (Student's t test, *P* value < 4 × 10^−4^). **B)** Immunoblot analysis demonstrating approximately equal ZPU1 content in strains analyzed in panel A. Three independent isolates of strains 808 and 830 were included in the analysis. “W64A” is a total soluble endosperm extract from non-mutant maize included as a positive control.

The yeast strains were grown in rich galactose medium to mid-logarithmic phase, then total soluble extracts were assayed for hydrolytic activity using the aforementioned Red Pullulan or solubilized amylopectin as substrate (Fig. 5A). Hydrolysis of amylopectin was measured by enzymatic detection of new reducing ends. In all instances, product formation was linear with time, and activity values were linear with extract volume. As expected, extracts of control strain 833 devoid of any exogenous enzyme did not exhibit detectable activity with either test substrate. Strain 806, containing only ISA1, was active with amylopectin but not Red Pullulan, consistent with the previously demonstrated substrate specificity for a recombinant form of this enzyme (Rahman et al. 1998). Strain 808, containing only ZPU1, catabolized Red Pullulan and amylopectin. These single enzyme controls established that any activity with the pullulan model substrate results from ZPU1. When both ISA1 and ZPU1 were present, in strain 830, the level of activity with Red Pullulan was significantly elevated, approximately 1.7-fold, compared to when ZPU1 alone was present (*P* value < 4 × 10^−4^).

The possibility that variation in ZPU1 activity in the presence or absence of ISA1 might be due to different levels of protein accumulation was tested by immunoblot analysis (Fig. 5B). Soluble extracts from 3 independent isolates of strains 808 or 830, derived from independent integrations into the yeast genome, were probed with anti-ZPU1 serum. Based on the immunoblot signal, the level of ZPU1 normalized to total protein was approximately equal in the strains containing or lacking ISA1. Taken together, these data indicate that ISA1 stimulates the enzymatic activity of ZPU1 toward Red Pullulan.

### ZPU1 activity in *su1-Ref* suppressor lines

Maize suppressor lines Wpse2 and Wpse3, termed pseudo-starchy in previous publications, are *su1-Ref* inbreds that were developed by recurrent selection (Tracy and Chang 2007; De Vries et al. 2016). These lines contain the W578R missense mutant form of ISA1 that prevents activity of any ISA complex (Dinges et al. 2001). The recurrent selection process began with cross-pollination of a collection of homozygous *su1-Ref* lines in undefined genetic backgrounds. In each cycle, plants were self-pollinated to make potential suppressor alleles homozygous, and kernels were visually inspected for a more normal appearance. Fifteen percent of the best-scoring seeds were then divided into subpopulations for cross-pollination in the next round of selection. After multiple rounds, the kernel appearance had returned to normal (De Vries et al. 2016). Inbred seed was derived from the normal-appearing kernels by repeated rounds of self-pollination, yielding suppressor lines Wpse2 and Wpse3. As expected from the normal visual appearance of the kernels, phytoglycogen content in the suppressed lines had returned to very low levels (Tracy and Chang 2007; De Vries et al. 2016).

Wpse2 and Wpse3 were confirmed as *su1-Ref* homozygotes by sequence analysis of a PCR-amplified fragment of genomic DNA. Both lines were homozygous for the T to C transition that constitutes *su1-Ref* (Table 1), ruling out the possibility that contaminating wild-type pollen might have entered the breeding population during recurrent selection. Further, crosses of Wpse2 or Wpse3 to standard *su1-Ref* lines resulted in the low-starch, high phytoglycogen accumulation phenotype typical of *su1-Ref* mutants. This indicates the suppressor alleles are extragenic and recessive, and confirms that all lines are homozygous for *su1-Ref*.

**Figure 6.**
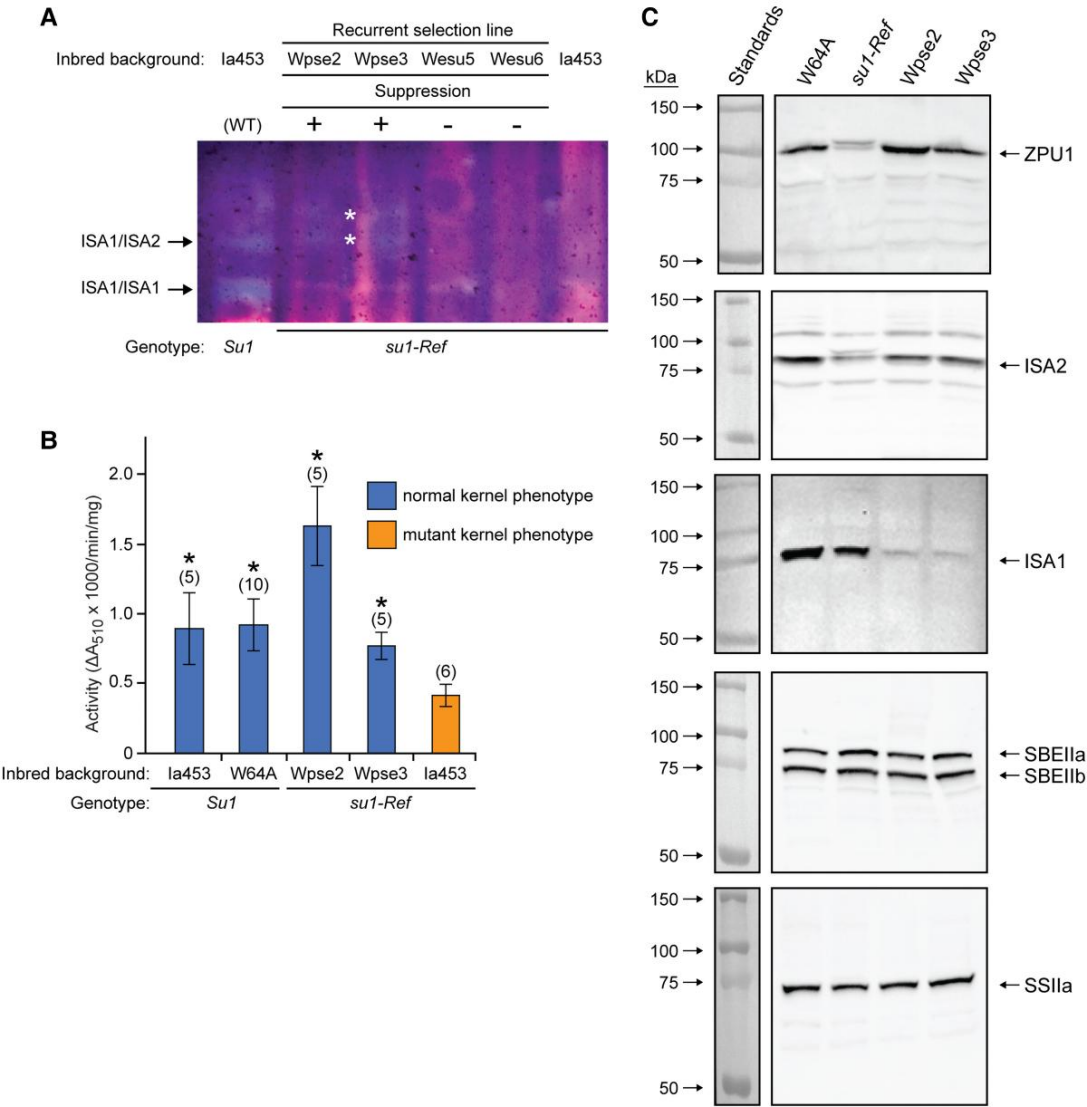
Enzyme activities and protein content in suppressor maize lines homozygous for *su1-Ref*. **A)** In-gel enzyme activity assay. Equal amounts of soluble endosperm extract were fractionated by native-PAGE, and then, proteins were transferred to a starch-containing gel in native conditions conducive to enzymatic activity. Staining the starch gel with iodine revealed light blue bands resulting from defined isoamylase activities present in the wild-type control (WT) and absent from congenic *su1-Ref* mutants (Kubo et al. 2010; Trimble et al. 2016). Four homozygous *su1-Ref* inbreds from the recurrent selection were compared (De Vries et al., 2016), 2 of which had reverted to a near-normal kernel phenotype (+) and 2 that retained the unsuppressed *su1-Ref* phenotype (−). ISA1/ISA1 homodimer activity is not restored in the suppressed lines. The state of the ISA1/ISA2 heteromultimers in the suppressed lines Wpse2 and Wpse3 cannot be discerned because they are masked by unidentified starch hydrolytic activities (marked by white asterisks). **B)** ZPU1 activity in total soluble endosperm extracts. Comparison is made between the unsuppressed *su1-Ref* mutant in the Ia453 genetic background (mutant kernel phenotype) and suppressed *su1-Ref* recurrent selection lines Wpse2 and Wpse3 that exhibit normal glucan storage and kernel phenotype. Non-mutant inbreds (*Su1*) are included as controls. Values are average ± standard deviation from the indicated number of biological replicates. Replicates are individual endosperms from a single ear of the indicated homozygous genotype. Asterisks (*) indicate significant difference from unsuppressed *su1-Ref* endosperm (mutant kernel phenotype) (Student's *t* test, *P* value < 10^−5^). **C)** Immunoblot analysis. Equal amounts of total soluble endosperm extract from the indicated maize lines were fractionated by SDS-PAGE and probed with antibodies specific for the indicated proteins.

Isoamylase activity was examined in the suppressor lines by zymogram assay with amylopectin as the substrate. The ISA1/ISA1 homodimer activity that is normally missing from *su1-Ref* mutants is still missing in the suppressed lines (Fig. 6A). This confirms that the suppressor alleles do not restore normal ISA1 activity. The state of the ISA1/ISA2 heteromultimers was obscured by co-migrating hydrolytic activities (Fig. 6A) (Supplementary Fig. S5). Considering the loss of the ISA1/ISA1 homomultimer, however, most likely none of the ISA activities have been restored in the suppressor lines.

ZPU1 activity in total endosperm extracts from the suppressed *su1-Ref* lines was measured by the hydrolysis of Red Pullulan. Wpse3 exhibited ZPU1 activity comparable to that seen in non-mutant standard, and activity in Wpse2 was substantially elevated above the wildtype level (Fig. 6B). Thus, the effect of *su1-Ref* that causes reduced ZPU1 activity has been suppressed in Wpse2 and Wpse3. Restored ZPU1 activity was also evident in the zymogram assay (Supplementary Fig. S5). ZPU1 protein content in Wpse2 and Wpse3 was approximately equal to that in wildtype strains (Fig. 6C) rather than the reduced level typical of *su1-Ref* endosperm. The electrophoretic mobility shift of ZPU1 seen in *su1-Ref* mutants was not evident in the suppressor lines. Thus, the effect(s) of *su1-Ref* that change the electrophoretic mobility and abundance of ZPU1 have also been suppressed in Wpse2 and Wpse3.

### Other starch-modifying activities in *su1-ref* suppressor lines

One hypothesis to explain suppression of the *su1-Ref* defect in ISA1 is a change in the ISA2 component of the ISA1/ISA2 heteromultimeric complex. The sequence of the ISA2 coding region in the suppressed lines, however, was found to match precisely the non-mutant allele present in inbred Ia453. Immunoblot analysis revealed that ISA2 accumulates at the non-mutant level in the suppressed lines (Fig. 6C). Thus, a change in ISA2 is not likely to be responsible for suppressing the effects of the *su1-Ref* mutation.

ISA1 levels were also measured by immunoblot analysis in the suppressed lines. These were reduced substantially compared to the level in unsuppressed *su1-Ref* lines (Fig. 6C). This rules out overexpression of the ISA1-W578R mutant protein as an explanation for suppression of the mutant phenotype. The reason that the suppressor alleles result in reduced ISA1 steady-state level remains to be determined.

Protein levels of 3 other starch biosynthetic enzymes were compared between the suppressed and unsuppressed *su1-Ref* lines, specifically BEIIa, BEIIb, and SSIIa (Fig. 6C). None of these proteins was changed in steady-state abundance by the alleles that suppress *su1-Ref*. Thus, the effects of the suppressor alleles on ISA1, ISA2, and ZPU1 protein abundance are not part of a general response that regulates all starch metabolism enzymes.

## Discussion

### Physical interactions among ZPU1, ISA1, and ISA2

This report provides 2 lines of evidence that ZPU1 and ISA1 can interact physically in quaternary assemblies, specifically (1) reconstitution of GAL4 function in yeast dependent on ZPU1/ISA1 binding, and (2) immunoprecipitation of ZPU1 by antibodies specific for ISA1. In addition, indirect evidence consistent with ZPU1–ISA1 interaction was provided by the complementary observations that when ISA1 is missing or mutated in endosperm cell extracts, the catalytic activity of ZPU1 is decreased, and when ISA1 is present in yeast cell extracts, ZPU1 activity is stimulated. Further indirect evidence for interaction comes from the observation that the state of the ZPU1 protein, as indicated by mobility in SDS-PAGE, is altered when ISA1 is absent from endosperm extracts or changed by various single amino acid substitutions. Assembly of ISA1 and ISA2 in heteromultimeric complexes in chloroplasts is well established, and the results of the current study raise the possibility that a third GH13 protein, ZPU1, also associates physically with ISA1 and ISA2.

Whether the observed interaction between ZPU1 and ISA1 is direct or mediated by an intermediary protein has not been definitively established. Immunoprecipitation does not distinguish between these 2 possibilities. In vivo protein–protein interaction was tested in yeast cells that do not contain any maize protein other than the GAL4-ZPU1 and GAL4-ISA1 fusion proteins. Therefore, ZPU1 and ISA1 are capable of forming a complex in the absence of any other factor from maize endosperm, and this is most likely explained by a direct interaction between the 2 proteins. Indirect interaction is likely for the connection between ISA2 and ZPU1. That combination did not generate a positive signal in the in vivo protein–protein interaction test, even though both proteins yielded positive results with other binding partners. Interaction between ZPU2 and ISA2 was indicated, however, by the fact that eliminating ISA2 from endosperm extracts caused a reduction in ZPU1 enzymatic activity. These results may be explained by a direct interaction of ZPU1 with ISA1 within a complex that also contains ISA2. Removal of ISA2 from that complex could thus result in an effect on ZPU1 catalytic capacity. The result suggests further that ZPU1 acts specifically with the ISA1/ISA2 heteromultimer(s), because the ISA1/ISA1 homodimer remains in *isa2-* null mutants, yet ZPU1 activity is nonetheless reduced significantly when ISA2 is absent.

The association between ZPU1 and the ISA1/ISA2 complexes is apparently not stable through native gel electrophoresis. ISA1 stays tightly associated with itself as well as with ISA2 through native-PAGE, whereas the active forms of ZPU1 and the ISA complexes exhibit distinct mobilities in native-PAGE zymograms. Relatively weak interaction between ZPU1 and ISA1 was evident in the in vivo protein–protein interaction test, from the fact that the ISA1-ZPU1 combination did not consistently activate all 3 reporter genes. This is in contrast to the results with ISA1 and ISA2, which repeatedly showed robust positive signals in the stringent selection of the two-hybrid test. Further, ZPU1 has not previously been reported to co-purify with the ISA1 homodimer or ISA1/ISA2 heteromultimer complex. This does not rule out that ISA and ZPU1 interact directly in vivo because positive signals in co-immunoprecipitation and yeast two-hybrid tests may result from relatively low-affinity interactions, whereas co-purification and co-migration in native-PAGE may require higher affinity between the binding partners.

Further investigation is needed to determine whether the binding of ZPU1 to an ISA1 homomeric complex or an ISA1/ISA2 heteromultimer is a required aspect of the ZPU1 function that affects starch and phytoglycogen content. The results are consistent, however, with the hypothesis that the role of α-(1→6)-glucosidases in stimulating the crystallization of α-polyglucans depends at least in part on their association in quaternary complexes. The evolutionary sources of the PUL and ISA genes in plants and green algae were glycogen-accumulating eubacteria, not starch-producing organisms. Learning how the functions of these proteins changed in eukaryotes to affect starch formation, as opposed to their progenitor function in glycogen catabolism, will further our understanding of how land plants arose and spread on Earth. Evolution of substrate specificity is involved (Cenci et al. 2013; Kobayashi et al. 2016), however, another distinction between plant forms and their progenitors is assembly into complexes. Direct testing for potential quaternary structure of ISA or PUL in eubacteria has been limited, however, ISA from *Pseudomonas amylodermosa* was shown to be a monomer (Yokobayashi et al. 1970), and this is generally presumed to apply throughout the eubacterial kingdom. In contrast, ISAs required for normal levels of starch accumulation have repeatedly been found to exist in quaternary assemblies. In one example, a rare starch-accumulating cyanobacterium, strain CLg1, contains a second gene encoding ISA in addition to the *glgX* gene present in essentially all glycogen-accumulating eubacteria. The second gene, *glgX2*, produces an ISA enzyme that apparently exists as a trimer, as determined by size exclusion chromatography, and mutation of this enzyme causes a major decrease in starch content (Cenci et al. 2013). An archaeal ISA gene involved in trehalose metabolism, *TreX*, produces a protein that switches between a dimeric and tetrameric state, and this conversion alters the catalytic properties of the enzyme from a hydrolase to a glucanotransferase (Park et al. 2008). In the case of plants and green algae, as noted, ISA1 and ISA2 are well established to exist in heteromeric complexes that are required for normal starch accumulation.

The data reported here raise the possibility that as PUL evolved from a progenitor catabolic function into a form that modifies precursor α-polyglucans such that they can crystallize, it acquired the ability to form heteromeric complexes, including other α-(1→6) glucosidases in the GH13 family, namely ISA1 and ISA2. ISA and PUL proteins all possess substrate-binding channels where glucan chains can associate, as well as other likely glucan-binding structures (Katsuya et al. 1998; Song et al. 2010; Sim et al. 2014; Moller et al. 2015). The assembly of three different GH13 proteins together could affect how they interact with precursor substrates, and this could be different than for any such enzyme acting on its own as a monomer. Such a mechanism could also explain the stimulatory effect of ISA1 and ISA2 on ZPU1 catalytic efficiency.

### Multiple ZPU1 electrophoretic mobility forms

In addition to affecting the enzymatic activity of ZPU1, the presence or absence of ISA1 also changed the way that ZPU1 migrates in SDS-PAGE. This further supports the functional interaction between the 2 GH13 proteins and is consistent with a direct interaction between them. Two observed migratory forms of ZPU1 are present in non-mutant endosperm, and the relative abundance of each depends on the state of ISA1. The nature of the change in ZPU1 electrophoretic mobility is not known. One possible explanation is a failure in protein targeting to the plastid so that the transit peptide remains in a large proportion of the ZPU1 primary translation products when ISA1 is altered or absent. Another possibility is post-translational modification that affects mobility in SDS-PAGE. A third consideration is possible changes in protein folding. The ZPU1 homolog from spinach leaves has the unusual property of spontaneously folding into variant structures that separate on isoelectric focusing columns (Henker et al. 1998; Renz et al. 1998). Purified apparent charge variants from such columns spontaneously refold into each of the other forms that separate again during a second round of isoelectric focusing. Whether such a protein folding property could affect mobility in SDS gels is unknown, but this unusual property of a ZPU1 homolog should be noted as potentially important in understanding how the multiple DBEs act in concert.

The unusual effect of *su1-Ref* on another protein's mobility in SDS-PAGE was also seen for ISA2 (Kubo et al. 2010). Again, the molecular explanation for the variant mobility form is unknown, but most likely, this effect of *su1-Ref* on 2 different proteins results from a common mechanism.

### Suppression of phytoglycogen accumulation in *su1-ref* mutants

Maize Wpse2 and Wpse3 are separately derived inbreds that are homozygous for the *su1-Ref* amino acid substitution mutation W578R and accordingly lack ISA activity, yet accumulate near-normal levels of starch and very low levels of remnant phytoglycogen compared to typical, unsuppressed *su1-Ref* lines. The nature of the suppressing alleles is unknown, so the molecular explanation for why the phytoglycogen-accumulation phenotype is suppressed remains to be determined. ZPU1 activity is restored to normal or elevated levels in the suppressed lines, and this association is consistent with ZPU1 partially overlapping in function with the ISA1/ISA2 complexes regarding α-polyglucan crystallization. This observed correlation does not prove that ZPU1 activity is the cause of the glucan storage phenotype, although that is a possible explanation. Nonetheless, association between suppression of *su1-Ref* and restoration of ZPU1 activity provides further evidence of a functional relationship between the α-(1→6)-glucosidase activities of the ISA1/ISA2 complexes and ZPU1, i.e. between the separately acquired and separately conserved GH13_11 and GH13__13 enzymes.

The reason that the suppressor alleles result in reduced steady-state level of the ISA1-W578R protein remains to be determined. The *su1-Ref* mutation reproducibly results in a substantial decrease in the level of mutant ISA1 protein that accumulates compared to the non-mutant form (Kubo et al. 2010) (Fig. 6C). Starting from this reduced level, the suppressor alleles present in Wpse2 and Wpse3 cause a further decrease in the steady-state level of ISA1-W578R. These changes likely result at least in part from altered rates of protein turnover. This is known to be the case for ISA2, from the observation that mutations that entirely delete ISA1 expression also result in the complete absence of ISA2 protein (Kubo et al. 2010).

## Materials and methods

### Plant materials

Mutant alleles in the W64A inbred background (Table 1) were backcrossed a minimum of 5 times to the non-mutant standard. At each generation, heterozygous plants containing *su1-*mutant alleles were identified by self-pollination or test crosses to *su1-Ref* homozygotes, and those plants were simultaneously crossed to standard for the next backcross generation. After the fifth backcross, sibling heterozygotes were crossed to generate homozygous mutant kernels. Introgression of *isa2-339* or *zpu1-204* into the W64A background proceeded similarly, except that heterozygous plants were identified by PCR analysis of leaf genomic DNA, as previously described (Dinges et al. 2003a; Kubo et al. 2010). The same procedure was used for introgression of *su1-Ref* or *isa2-339* into the Ia453 inbred background (De Vries and Tracy 2016). The *su1-Ref* inbred P39 and a non-mutant conversion line in that background were previously described (Tracy et al. 2000). Lines in the A632 inbred background (Table 1) were described by Trimble et al. (2016). Recurrent selection lines were generated as described (Tracy and Chang 2007; De Vries et al. 2016).

For biochemical analyses, homozygous plants were field-grown in Ames, IA or Madison, WI. Kernels were harvested 20 days after pollination, flash frozen in liquid nitrogen, and stored at -80 ℃ until use.

### Endosperm extraction

Tissue extractions were performed on ice. Total soluble endosperm extracts were prepared from 5-10 endosperms dissected from immature kernels harvested 20 days after pollination. These were ground in a mortar and pestle in 2 mL of 50 mm Tris-acetate, pH 7.5, 5 mm MgCl2, 1 mm DTT, 1 mm PMSF, 0.15% Tween-20, and 1X protease inhibitor cocktail (Sigma no. P-2714). Extracts were centrifuged at full speed in a microfuge for 10 min. Supernatants were passed through a 0.45 *μ*m syringe tip filter, then saved as the total soluble extract. Protein concentration in the extracts was measured by the Bradford assay. These extracts were used for in-gel visualization of enzyme activity (zymograms), immunoblot analysis, and immunoprecipitation.

Total soluble endosperm extracts for enzyme activity assays were prepared as follows. Three endosperms were suspended in 500 *µ*L of 50 mm HEPES, pH 7.4, 5 mm MgCl2, 5 mm DTT, and homogenized in a 1.5-mL tube using a disposable pestle. The samples were then centrifuged at maximum speed in a microfuge for 5 min at 4 °C. To remove phytoglycogen from extracts of *su1-*mutant endosperm, 30 *µ*L of a 50% (w/v) solution of polyethylene glycol (Sigma no. 202444) was added to 270 *µ*L of the supernatant, and the solution was centrifuged again at maximum speed for 5 min at 4 °C. PEG treatment was also applied to extracts that lack phytoglycogen. The resulting supernatant was immediately used in the enzyme assay.

### α-(1→6)-glucosidase enzyme activity assays

Hydrolysis of α-(1→6) linkages in pullulan was measured using the artificial substrate Red Pullulan (Megazyme catalog no. S-RPUL). Assays in a volume of 225 *μ*L contained 88.9 mm potassium phosphate, pH 6.0, 167 mm KCl, 0.67% mg Red Pullulan, and 300–400 *μ*g of total soluble cell extract from either yeast or maize endosperm. These reactions were prepared by mixing 75 *μ*L of 2% Red Pullulan in 0.5 m KCl, 100 *μ*L of 200 mm potassium phosphate, pH 6.0, and 50 *μ*L of cell extract. Reactions were incubated for 60 min at 37 ℃, then 375 *μ*L of 100% ethanol was added to stop the reaction and precipitate undigested Red Pullulan polymer while leaving released maltooligosaccharides in solution. After 10 min at room temperature, the reactions were clarified by centrifugation at 1360*×g* for 10 min. Absorbance of the supernatant at 510 nm was measured as a quantitative indication of the number of alpha-1,6-glycoside bonds hydrolyzed.

Hydrolysis of α-(1→6) linkages in amylopectin was measured by the formation of new reducing ends detected by reaction with 3,5-dinitrosalicylic acid (DNSA). Assays in a volume of 150 *μ*L contained 2.5% maize amylopectin (Sigma no. 10120), 25 mm sodium phosphate, pH 6.0, 5 mm MgCl_2_, and 300–400 *μ*g of clarified yeast extract. After 0, 30, or 60 min at 37 ℃ the reactions were terminated by the addition of 450 *μ*L DNSA solution. Color development was achieved by boiling the reactions for 5 min, incubation on ice for 5 min, and centrifugation for 5 min at full speed in a microfuge. Maltose (100–500 nmol) was used as a standard curve. A minimum of 3 biological replicate analyses were performed for each sample. All reported assays were linear with time over 60 min. DNSA solution was prepared by dissolving 1 g of DNSA in 20 mL of 2 m NaOH. In a separate beaker, 30 g of sodium potassium tartrate was dissolved in 50 mL of H_2_O. The 2 components were mixed, heated until homogeneous, brought to 100 mL with H_2_O, and stored in an amber bottle at 4 ℃.

In-gel activity assays were performed as previously described (Colleoni et al. 2003; Kubo et al. 2010). Briefly, total soluble extracts (50 *μ*g) were fractionated by native-PAGE, and then proteins in those gels were electrophoretically transferred to a polyacrylamide gel impregnated with starch in buffer conditions conducive to enzyme activity. After staining with iodine solution, colored bands indicated positions of enzymes that alter the starch structure and thus change the starch iodine-complex absorption spectrum from that of the background. DBE activities in these assays result in light blue bands over a dark purple background.

### Immunological methods

The preparation of anti-ZPU1 total crude serum was described previously (Beatty et al. 1999). The antigen used to elicit anti-ZPU1 was a recombinant protein containing residues 160–918 relative to the primary translation product (Supplementary Table S1). Anti-ISA1 and anti-ISA2 affinity-purified IgG fractions were prepared as previously described (Kubo et al. 2010). These sera were raised against peptides specific to each protein. IgG that binds those peptides was purified by affinity chromatography. Specificity of the serum or IgG fractions was confirmed by immunoblot analysis of non-mutant endosperm extract and extracts from the corresponding null mutant, i.e. *su1-4582*, *isa2-339*, or *zpu1-204* (Supplementary Fig. S4). Antisera used for the detection of SBEIIa and SBEIIb, or for SSIIa, were previously described (Hennen-Bierwagen et al. 2008).

SDS-PAGE and immunoblotting procedures followed standard protocols. Approximately 20–40 *μ*g of total soluble endosperm extract was applied to each lane of the gel. Anti-ISA1 IgG was exposed to the blots at a dilution of 1/1,000, anti-ZPU1 serum was used at a dilution of 1/5,000, and anti-ISA2 IgG was used at a dilution of 1/1,000.

Immunoprecipitation utilized protein A-Sepharose 4B conjugate (Invitrogen no. 101041). A bed volume of 400 *μ*L was washed extensively in standard phosphate-buffered saline (PBS) by suspending the beads in 1 mL of wash solution, inverting several times, centrifuging at 500*×g* for 2 min, and then discarding the supernatant. The beads were incubated with anti-ISA1 IgG (350 *μ*L antibody plus 50 *μ*L PBS) with periodic gentle mixing on ice for 60 min, then washed 4 times in PBS. The beads from the final wash were suspended in 400 *μ*L of PBS, and 200 *μ*L of the slurry was distributed into 1.5 mL polypropylene tubes. To each tube was added 100 *μ*L of clarified endosperm extract (∼500 *μ*g total protein) and 400 *μ*L PBS. The beads were incubated on ice for 60 min with periodic gentle agitation, then washed 5 times in PBS supplemented with 1% NP-40. After the final wash, the beads were boiled in 100 *μ*L of 1X SDS-PAGE loading buffer (62.5 mm Tris-HCl, pH 6.8, 10% glycerol, 2% SDS, 5% β-mercaptoethanol, 0.025% bromophenol blue) for 10 min. After centrifugation, 20 *μ*L of each eluate was applied in duplicate to an SDS-PAGE gel. One set of gel lanes was probed in standard immunoblot analysis with anti-ISA1 IgG, and the second set was probed with anti-ZPU1 serum.

### Growth media for yeast

Complete media contained 2% Bacto Peptone, 1% Bacto Yeast Extract with 2% glucose (YPD) or 2% galactose (YPGal). Minimal media contained 2% glucose, 0.67% Yeast Nitrogen Base without amino acids (Difco no. 0919-15-3), and 0.096% synthetic dropout media supplements lacking Leu and Trp (Sigma no. Y0750), lacking Leu, Trp, and His (Sigma no. Y2146), or lacking Leu, Trp, His, and Ade (Sigma no. Y2021). AbA (Takara no. 630499) was added to solid minimal media at 0.6 mg/mL.

### In vivo protein–protein interaction tests

Codon-optimized open reading frames (ORF) that encode ZPU1 or ISA1 attached to flanking sequences for use in restriction/ligation cloning or Golden Gate assembly (Werner et al. 2012) were synthesized commercially (Supplementary Fig. S2). The primary structure range included in each ORF is detailed in Supplementary Table S1. Restriction fragments coding for ISA1, ISA2-S, ISA2-L, or ZPU1 were excised from the synthetic plasmids as *Nde*I/*Bam*HI fragments (Supplementary Fig. S2) and cloned into plasmid pGBKT7 of the Takara Bio USA Matchmaker Gold Yeast Two-Hybrid System (Catalog no. 630443) to create GAL4 binding domain fusions. The same fragments were also cloned into plasmid pGADT7 from the Matchmaker Gold system to create GAL4 activation domain fusions.

The binding domain fusion plasmids were transformed into yeast (*Saccharomyces cerevisia*e) strain Y2HGold, and the activation domain plasmids were transformed into strain Y187, both from the Matchmaker Gold system. None of the binding domain plasmids by themselves activated the AbA-resistance gene of the host strain Y2HGold. Strains were mated pairwise, and diploids were selected by complementing auxotrophies on -Trp, -Leu minimal medium. Plating the mating mixtures on -Trp, -Leu, -His minimal medium selected for diploids that had activated the histidine prototrophy marker, and plating on -Trp, -Leu, -His, -Ade, +AbA simultaneously selected for 3 separate reporter gene activities.

### Yeast strain construction

Promoters and transcriptional terminators were added to the ORFs encoding ISA1 or ZPU1 by Golden Gate assembly to form complete transcription units (TU) (Supplementary Table S3). The TUs encoding ISA1 or ZPU1 were individually integrated into the yeast genome. The ISA1 and ZPU1 TUs also were combined into a TU array plasmid (Supplementary Table S4) and then integrated together into the yeast genome. A TU array plasmid lacking any exogenous coding sequence was constructed (Supplementary Table S4) and used for the construction of a congenic control strain devoid of maize genes.

The genotypes of the yeast strains used in this study are specified in Supplementary Table S2. Host strains 506.1 and 556.1 were developed by Pfister et al. (2022). In both host strains, the endogenous genes coding for glycogen synthase (*GSY1*, *GSY2*), glycogenin (*GLG1*, *GLG2*), glycogen branching enzyme (*GLC3*), and maltase (*MALx2*) are inactivated. Strain 506.1 also contains a disruption of the gene encoding glycogen phosphorylase (*GPH1*). The functional copy of the glycogen debranching enzyme gene (*GDE1*) in strain 506.1 was the target site for insertion of the TU encoding ISA1, forming strain 806, or the negative control construct that lacks any maize gene, forming strain 833. Host strain 556.1 contains a disruption of *GDE1* and a functional copy of the gene encoding glycogen phosphorylase (*GPH1*). *GPH1* in strain 556.1 was the target site for insertion of the TU encoding ZPU1, forming strain 808, and the TU array encoding both ZPU1 and ISA1, forming strain 830. In summary, the experimental yeast strains are congenic and contain disruptions of all glycogen metabolism genes and maltase genes. They vary by inclusion of ISA1, ZPU1, both ISA1 and ZPU1, or are devoid of maize enzymes.

### Yeast cell growth and extraction

YPD pre-growth cultures were inoculated from a single colony and grown overnight at 30 ℃. Cells were harvested by centrifugation and then washed once in an equal volume of YPGal medium. Cells were added to YPGal cultures so that A_600_ ≅0.3, then grown at 30 ℃ with shaking for 5.75 h. Cells were collected by centrifugation at 7000 rpm for 5 min, then washed once with H_2_O, and collected by centrifugation. The wet weight of the cell pellet was recorded; then, the cells were stored at −80 ℃ until further use.

For enzyme assays, cell pellets from 40 mL cultures were suspended in 300 *μ*L of ice-cold 50 mm sodium phosphate, pH 6.0, in a 1.5-mL polypropylene centrifuge tube. A 200 *μ*L volume of acid-washed, 400-*μ*m glass beads was added, and then, the cells were homogenized in a Spex 1600 MiniG Tissue Homogenizer at maximum speed for 4 treatments of 2 min each, with cooling for 2 min between each treatment. Homogenates were then centrifuged at maximum speed at 4 °C in a microfuge, and the supernatants were collected and designated as the total soluble yeast cell extract. Protein concentration in the extracts was determined by Bradford assay.

### Genomic sequencing

Oligonucleotide primers used for PCR amplification of maize genomic DNA are specified in Supplementary Table S5. The *isa2* and *su1* loci were analyzed from lines W64A, Ia453, Wpse2, and Wpse3. The gene models for these 2 loci are Zm00001eb287400 and Zm00001eb17459, respectively, from the B73 reference genome sequence Zm-B73-REFERENCE-NAM-5.0 (www.maizegdb.org). The region of the *su1* genomic locus containing the *su1-Ref* allele was amplified from embryo genomic DNA prepared as described (Lappe et al. 2018) using primers su1W-RF and su1W-RR. Amplified fragments were sequenced in both directions using the same primers. The region of the *isa2* locus containing the complete coding sequence was amplified in 4 overlapping fragments using primer sets ISA2F1/ISA2R1, ISA2F2/ISA2R2, ISA2F3/ISA2R3, and ISA2F4/ISA24R. All 4 amplified fragments were sequenced in both directions using the same primers.

### Statistical analyses

Pairwise comparison of data sets to determine *P* values used the Student's t test function in Microsoft Excel with options for two-tailed distributions and two-sample comparisons with equal variance.

### Accession numbers

Sequence data mentioned in this article can be accessed from the maize genome database (MaizeGDB; https://www.maizegdb.org) within the gene models noted in Table 1.

## Supplementary Material

kiaf417_Supplementary_Data

## Data Availability

The data underlying this article are available in the article and in its online supplementary material.

## Supplementary data

The following materials are available in the online version of this article.

**Supplementary Table S1.** Maize proteins expressed in yeast.

**Supplementary Table S2.** Yeast strain genotypes.

**Supplementary Table S3.** Transcription units.

**Supplementary Table S4.** Transcription unit array plasmid and control plasmid.

**Supplementary Table S5.** Oligonucleotide primers used for amplification of maize genomic DNA.

**Supplementary Figure S1.** Full gel immunoblot data accompanying Fig. 2.

**Supplementary Figure S2.** Synthetic gene sequences codon optimized for expression in yeast.

**Supplementary Figure S3.** In vivo protein–protein interaction tests accompanying Fig. 3.

**Supplementary Figure S4.** Antibody specificity.

**Supplementary Figure S5.** In-gel enzyme activity assay of starch hydrolytic activities in recurrent selection inbreds.

## Dive Curated Terms

The following phenotypic, genotypic, and functional terms are of significance to the work described in this paper:

ISA1 Gramene: AT2G39930
ISA1 Araport: AT2G39930

ISA3 Gramene: AT4G09020
ISA3 Araport: AT4G09020

Zpu1 Gramene: Zm00001eb088740
Zpu1 Araport: Zm00001eb088740

starch CHEBI: CHEBI:28017

protein CHEBI: CHEBI:36080

amylopectin CHEBI: CHEBI:28057
