# Supplementary material for: Interactions of separately conserved α-(1→6) glucosidases that participate in maize endosperm starch biosynthesis
Source: Plant Physiol. 2025 Sep 23;199(2):kiaf417. doi: 10.1093/plphys/kiaf417 (PMC12516185; doi:10.1093/plphys/kiaf417)
Supplement: kiaf417_Supplementary_Data [file kiaf417_supplementary_data.pdf]

Supplementary Table S1. Maize proteins expressed in yeast

Protein name	Gene	Genomic identifier[a]	Residues[b]	Mass (kDa)
ZPU1	*zpu1*	Zm00001eb088740	80-962	84.9
ISA1	*su1*	Zm00001eb17459	50-789	83.4
ISA2-S	*isa2*	Zm00001eb287400	76-799	79.1
ISA2-L	*isa2*	Zm00001eb287400	34-799	83.7

[a] Gene model from wildtype inbred B73 reference genome sequence Zm-B73-REFERENCE-NAM-5.0 (www.maizegdb.org).

[b] Residue numbering refers to the primary translation product predicted by the indicated genomic identifier. The amino terminus for ISA1 proteins matches the known *in vivo* cleavage site, The ZPU1 amino terminus was chosen based on computational predictions for cleavage of the primary translation product during transport into chloroplasts.

Supplementary Table S2. Yeast strain genotypes

Strain	Genotype	Construction
506.1	*MATa MAL2-8C SUC2 his3Δ KanR ura3-52 malx2 glc3Δ gsy2Δ glg1Δ glg2Δ gph1Δ gsy1::pGAL1-glgC-TM-HA-tCYC1 bar1Δ XII-2::pCWP2-mCherry-tUPT7 GDE1*	Pfister et al., 2022
556.1	*MATa MAL2-8C SUC2 his3Δ KanR ura3-52 malx2 glc3Δ gsy2Δ glg1Δ glg2Δ gdb1Δ gsy1::pGAL1-glgC-TM-HA-tCYC1 bar1Δ XII-2::pCWP2-mCherry-tUPT7 GPH1*	Pfister et al., 2022
833	*MATa MAL2-8C SUC2 his3Δ KanR ura3-52 malx2 glc3Δ gsy2Δ glg1Δ glg2Δ gph1Δ gsy1::pGAL1-glgC-TM-HA-tCYC1 bar1Δ XII-2::pCWP2-mCherry-tUPT7 gde1::URA3*	Transformation of 506.1 with *Not*I fragment from pSB234
806	*MATa MAL2-8C SUC2 his3Δ KanR ura3-52 malx2 glc3Δ gsy2Δ glg1Δ glg2Δ gph1Δ gsy1::pGAL1-glgC-TM-HA-tCYC1 bar1Δ XII-2::pCWP2-mCherry-tUPT7 gde1::pCWP2-ISA1-tUPT7-URA3*	Transformation of 506.1 with *Not*I fragment from pSB230
808	*MATa MAL2-8C SUC2 his3Δ KanR ura3-52 malx2 glc3Δ gsy2Δ glg1Δ glg2Δ gdb1Δ gsy1::pGAL1-glgC-TM-HA-tCYC1 bar1Δ XII-2::pCWP2-mCherry-tUPT7 gph1::GAL1p-ZPU1-tUPT7-URA3*	Transformation of 556.1 with *Not*I fragment from pSB175
830	*MATa MAL2-8C SUC2 his3Δ KanR ura3-52 malx2 glc3Δ gsy2Δ glg1Δ glg2Δ gdb1Δ gsy1::pGAL1-glgC-TM-HA-tCYC1 bar1Δ XII-2::pCWP2-mCherry-tUPT7 gph1::pGAL1-ZPU1-tUPT7-pCWP2-ISA1-tUPT7-HygR*	Transformation of 556.1 with *Not*I fragment from pSB235

Supplementary Table S3. Transcription units.

Plasmid		5' Connector[b]	Transcription unit[c]			3' Connector[b]	Yeast Integration	
Name	Vector[a]		Promoter	ORF	Terminator		Marker	Site
pSB175	p144	ConL2	GAL1	ZPU1	UPT7	ConR3	*URA3*	*GPH1*
pSB230	p141	ConLS	CWP2	ISA1	UPT7	ConR1	*URA3*	*GDE1*

[a] Integration vectors used as cloning vectors for assembly of the ISA1 and ZPU1 transcription units were described by Pfister et al. (2022).

[b] Connector sequence blocks in the integration vectors provide *Bsa*I sites for Golden Gate assembly of the transcription units and *Bsm*BI sites for subsequent higher order assembly of the individual synthetic genes into transcription unit arrays. These sequence elements were described by Lee et al. (2015).

[c] Promoter and terminator elements were previously described (Pfister et al., 2022). Open reading frames encoding ZPU1 or ISA1 are specified in Supplementary Figure S2.

Supplementary Table S4. Transcription unit array plasmid and control plasmid. Cloning vectors and source plasmids are described by Pfister et al., (2022) or in Supplementary Table S3. Connector sequences were provided by Golden Gate part plasmids described by Lee et al. (2015).

Source Plasmid		Array Position								Yeast integration	
Name	Vector	1, ConLS-ConR1		2, ConL1-ConR2		3, ConL2-ConR3		4, ConL3-ConRE		Marker	Site
		Content	Source	Content	Source	Content	Source	Content	Source		
pSB234	p153	Spacer	p352	Spacer	p353	Spacer	p354	Spacer	p355	*URA3*	*GDB1*
pSB235	p328	ISA1	pSB230	Spacer	p353	ZPU1	pSB175	Spacer	p355	*Hyg*^{R}	*GPH1*

Supplementary Table S5. Oligonucleotide primers used for amplification of maize genomic DNA.

Primer name	Sequence
su1W-RF	5' CAA TTA GGT GGA TTA GTG 3'
su1W-RR	5' ATG AAA CTC TAA AGT GCG 3'
ISA2F1	5' ACC CTT TCC CAC CCG CGC 3'
ISA2R1	5' GGT CCG TCC CCG CCG CCA 3'
ISA2F2	5' ACA GCA AGA TCG CCA AGG 3'
ISA2R2	5' TCT TTG AAA GAA CAG GAT 3'
ISA2F3	5' TGG AAG CCA TAG CAT TTG 3'
ISA2R3	5' GCA TTG AAA CAG ATA TAC 3'
ISA2F4	5' ATC CTA CTA GCA ACT TTC 3'
ISA2R4	5' AAA CCA CAC GAG CTT CAT 3'

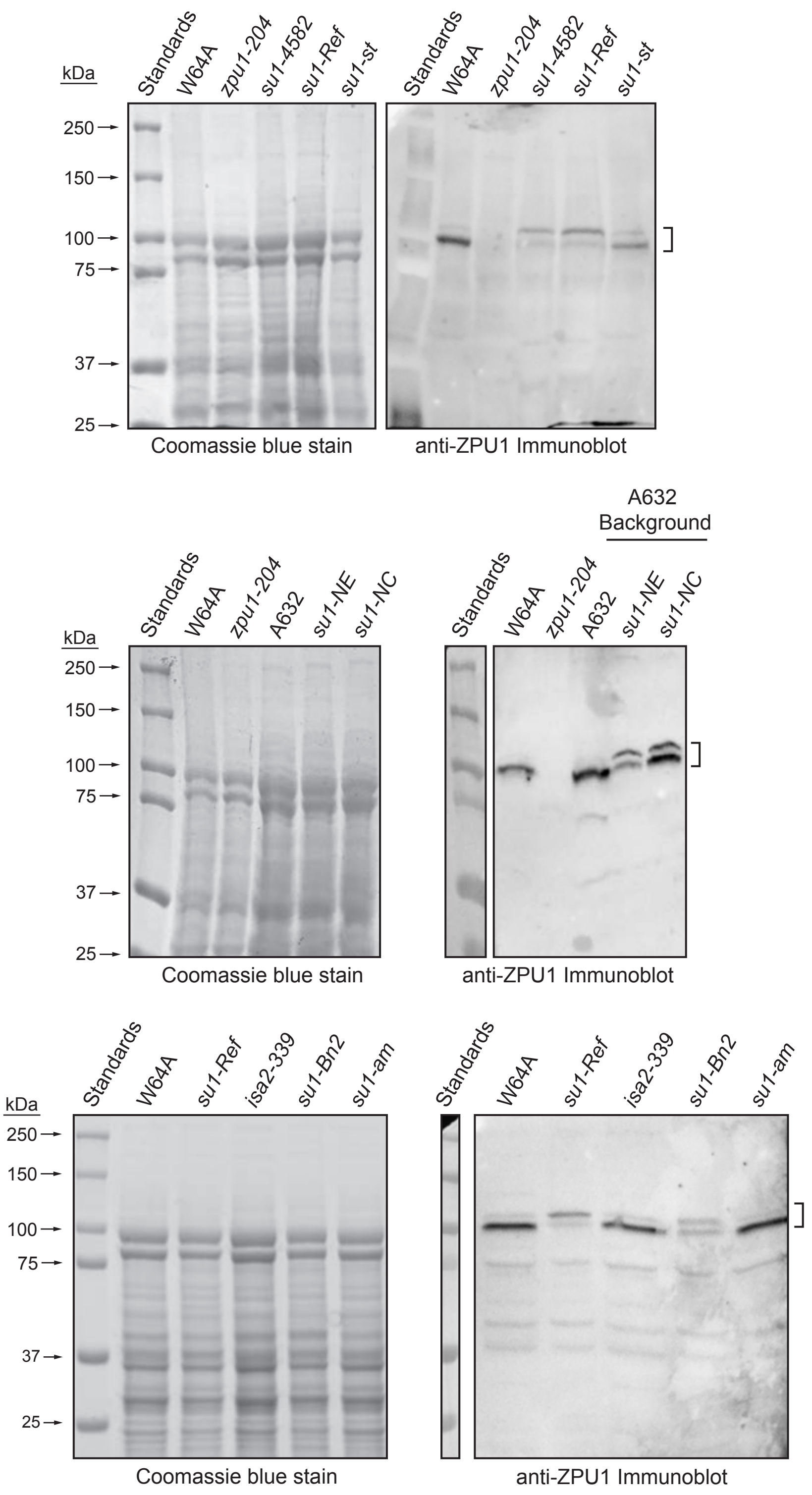

Supplementary Figure S1. Full gel immunoblot data accompanying Figure 2. Total soluble endosperm extracts (30 μg) from kernels harvested 20 DAP were fractionated by SDS-PAGE in duplicate gels and stained with Coomassie blue or probed with anti-ZPU1 antiserum. Brackets indicate ZPU1 bands.

>ZPU1

*Nde*I

GCATCGTCTCATCGGTCTCATATGGCACAGGGTTTTCTGCTGGATGCGCGTGCTTACTGGGTTACCAAAAGCCTGATTGCGTGGAACATCAGCGACC
AAAAGACCAGCCTGTTCCTGTACGCAAGCCGTAATGCGACCATGTGCATGAGCAGCCAAGATATGAAAGGTTATGACAGCAAGGTTGAACTGCAGCC
GGAAAACGATGGTCTGCCGAGCAGCGTGACCCAAAAATTTCCGTTCATTAGCAGCTACCGTGCTTTTCGTATCCCGAGCAGCGTTGACGTGGCAACC
CTGGTTAAGTGTCAGCTGGCAGTGGCGAGCTTCGATGCGCATGGTAATCGTCAAGACGTTACCGGTCTGCAGCTGCCGGGTGTGCTGGATGACATGT
TTGCATATACCGGTCCGCTGGGTACCATTTTCAGCGAAGAAGCGGTTAGCATGTACCTGTGGGCACCGACCGCGCAGGATGTTAGCGTGAGCTTTTA
TGACGGTCCGGCTGGTCCGCTGCTGGAAACCGTGCAACTGAATGAACTGAACGGTGTTTGGAGCGTGACCGGTCCGCGTAATTGGGAAAACCGTTAC
TATCTGTACGAAGTTACCGTGTATCACCAAACCACCGGTAACATTGAAAAATGTTTGGCTGCTGATCCGTACGCGAGAGGTCTGAGCGCTAATAGCA
CCCGTACCTGGCTGGTTGATATCAATAACGAAACCCTGAAGCCACTGGCTTGGGATGGTCTGGCAGCTGAAAAGCCGCGTCTGGATAGCTTTAGCGA
CATTAGCATCTATGAACTGCATATTCGTGATTTCAGCGCTCACGATAGCACCGTTGACTGCCCGTTTCGTGGTGGTTTCTGTGCATTTACCTTCCAG
GATAGCGTTGGTATCGAACATCTGAAAAAGCTGAGCGACGCGGGTCTGACCCATGTGCACCTGCTGCCGAGCTTTCAATTCGGTGGTGTTGATGACA
TTAAAAGCAACTGGAAGTGTGTGGATGAAATCGAACTGAGCAAACTGCCGCCGGGTAGCGACCTGCAACAGGCTGCAATTGTTGCTATCCAGGAAGA
AGATCCGTACAATTGGGGTTATAACCCGGTTGTGTGGGGTGTGCCGAAGGGTAGCTACGCTAGCAATCCGGACGGTCCGAGCCGTATTATCGAATAT
CGTCTGATGGTTCAAGCACTGAACCGTCTGGGTCTGCGTGTTGTGATGGATGTTGTGTACAATCATCTGTATAGCAGCGGTCCGTTTGCTATTACCA
GCGTTCTGGATAAAATCGTGCCGGGTTACTATCTGCGTCGTGACAGCAATGGTCAGACCGAAAACAGCGCGGCTGTTAATAACACCGCGAGCGAACA
CTTCATGGTTGACCGTCTGATCGTGGATGACCTGCTGAATTGGGCTGTTAACTACAAGGTGGATGGTTTTCGTTTCGACCTGATGGGTCATATTATG
AAAAAGACCATGATCCGTGCTAAAAGCGCACTGCAAAGCCTGACCATTGATGAACACGGTGTTGACGGTAGCAAGATCTACCTGTATGGTGAAGGTT
GGAACTTTGGTGAAGTGGCAGAAAATCAACGTGGTATTAACGGTAGCCAGCTGAATATGAGCGGTACCGGTATCGGTAGCTTTAATGATCGTATTCG
TGACGCGATCAACGGTGGTAGCCCGTTCGGTAATCCGCTGCAACAGGGTTTTAGCACCGGTCTGTTCCTGGAACCGAACGGTTTCTACCAGGGTAAT
GAAACCGAAACCCGTCTGACCCTGGCGACCTATGCTGATCATATTCAAATCGGTCTGGCGGGTAACCTGAAAGACTATGTTGTGATTAGCCACACCG
GTGAAGCTCGTAAGGGTAGCGAAATCCGTACCTTTGATGGTAGCCCGGTTGGTTACGCGAGCAGCCCGATTGAAACCATCAACTATGCTAGCGCACA
TGATAATGAAACCCTGTTCGACATTATCAGCCTGAAAACCCCGATGGATCTGAGCATTGACGAACGTTGCCGTATCAACCATCTGAGCACCAGCATG
ATTGCGCTGAGCCAAGGTATCCCGTTTTTCCACGCTGGTGATGAAATTCTGCGTAGCAAAAGCCTGGATCGTGACAGCTACGATAGCGGTGACTGGT
TTAATAAGATCGACTTCACCTATGAAACCAATAACTGGGGTGTTGGTCTGCCGCCGCGTGAAAAGAATGAAGGTAGCTGGCCGCTGATGAAGCCGCG
TCTGGAAAATCCGAGCTTTAAACCGGCTAAGCACGATATTATCGCAGCGCTGGATAAGTTCATCGACATCCTGAAGATCCGTTATAGCAGCCCGCTG
TTCCGTCTGACCACCGCAAGCGACATCGTTCAGCGTGTGCATTTCCACAACACCGGTCCGAGCCTGGTTCCGGGTGTTATTGTGATGAGCATCGAAG
ATGCACGTAATGATCGTCATGACATGGCGCAAATTGACGAAACCTTTAGCTGCGTTGTGACCGTTTTCAACGTGTGTCCGTACGAAGTGAGCATTGA
AATCCCGGATCTGGCTAGCCTGCGTCTGCAACTGCACCCGGTTCAAGTGAATAGCAGCGATGCGCTGGCTCGTCAAAGCGCTTATGACACCGCAACC
GGTCGTTTTACCGTTCCGAAGCGTACCGCTGCAGTTTTCGTGGAACCGCGTTGTTAAATCCTGAGACCTGAGACGGCATGGATCC

*Bam*HI

>ISA2-L

*Nde*I

GCATCGTCTCATCGGTCTCATATGATGCGTGCTGTGGCTCGCTCCTACCGTTACCGTTTTAGAACCGATGATGATGGCGTGGTGGATGTGGCTGTGG
CGGGCAAAGATGGTGACGCGGGTTACGTTGTGGCAATTGAAGCGCCGACCCATGGTCAACGTGGTGGTCTGGTGCTGCGTCCGGCTGGTAGCGGTGA
AGGTGTGCCGCTGGCACCAGCTGCTCCGGGTGGTGCTCTGGTTGCAGAACTGAGCTATGACGTGGCACGTGCGCCGTTTCATGTTAGCTTCACCCTG
GCTGATGCAATGGGTGCAGAAATCCGTACCCACCGTGGTACCAGCTTTCGTGTTCCGGTGGGTGTTGGTAGAGGTTGTCCGAGCCCGCTGGGTCTGA
GCCAGAGCAAAGACGGTGCAGCGAATTTTGCAGTGTACAGCAAAATTGCGAAGGGTATGGTTCTGTGTCTGTTCGGTGGTGGTGGTGGTGATGGTCC
GGCTCTGGAAATCGAACTGGACCCGTATGTTCATCGTACCGGTGATGTGTGGCACGTTAGCATGGAAAGCGTGGAAGGTTACGCGCGTTATGGTTTT
CGTAGCGGTCTGTTTGCTATGTTCGGTATTGATCGTCCGCTGCTGGACCCGTACGCAAAAGTGATCGGTGATTTCGTTGCGGGTGACAGCGTGGATG
AAGACGGTCTGGCTGTTCCGAGCATTCGTTGCCTGGCGAGCCTGAAGAATGCTCCGAACTACGATTGGGGTCGTGACAAACACCCGTGTCTGCCGCT
GGAAAAGCTGGTTGTGTATCGTGCGAATGTTGCTCTGTTCACCAAAGACCGTAGCAGCCGTCTGGCAGATAACGCTGCAGGTACCTTTAGCGGTATG
AGCGCGAAGGTTGAACATTTCCGTCACCTGGGTGTGAATGCTGTTCTGCTGGAACCGGTTTTTCCGTTCCATCAAGTGAAAGGTCCGTACTTTCCGT
ATCACTTTTTCAGCCCGATGAGCCTGTATAGCAGCGAATGCAGCAGCGTGAGCGCGATTAAAAGCATGAAGGACATGGTTAAGACCATGCATCGTAA
CGGTATCGAAGTGCTGCTGGAAGTTGTGTTCACCCACACCGCAGAAGGTGGTGCGGAATGTCAAATGATTAGCCTGCGTGGTATCGACGGTAGCAGC
TACTATATTGCTGATGGTATCGCAGGTTGCAAAGCGAGCGTGCTGAATTGTAACCATCCGGTTACCCAGAAGCTGATTCTGGATAGCCTGCGTCATT
GGGTTCTGGATTTTCACGTGGACGGTTTTTGCTTCATCAACGCTCCGTTCCTGGTTCGTGGTCCACGTGGTGAAGGTCTGAGCCGTCCGCCGCTGCT
GGAAGCTATTGCTTTTGACCCGGTTCTGAGCAAAACCAAGATTATCGCGGACCCGTGGAGCCCGCTGGATATCAGCAATGTGCAGTTTCCGTTCCCG
CACTGGAAGCGTTGGGCTGAAATGAACACCCGTTTTAGCATGGATGTTCGTAAATTCCTGAAGGGTGAAGCTCTGATCAGCGACCTGGCAACCCGTC
TGTGTGGTAGCGGTGATCTGTTCAGCAGCCGTGCACCGGCGTTTAGCTTCAATTACGTTAGCCGTAACAGCGGTCTGACCCTGGTGGACCTGGTTAG
CTTTAGCAGCGATGAACTGGCAAGCGAATTCAGCTGGAATTGCGGTGAAGAAGGTCCGAGCGAAAATAACGCGGTTCTGCAAACCCGTCTGCGTCAG
ATTCGTAACTTTCTGTTCATTCTGTTTATCAGCCTGGGTATCCCGGTGCTGAATATGGGTGACGAATGTGGTAACAGCGCGGCTGGTAGCACCAGCT
ATAAAGATCGTGGTCCGCTGAATTGGAAAGCTCTGAAGACCGCATTCGTGAAGGAAGTTACCGGTTTTATTAGCTTCCTGAGCGCGCTGCGTAGCCG
TCGTGCTGATATTTTTCAACGTTGCGAATTCCTGAAACTGGAAAACATCCATTGGTACGGTAGCGACCTGAGCGAACCGTGCTGGGAAGATCCGACC
AGCAATTTTCTGTGTCTGCACATCAACGCTGAACTGGATGAAAAGCTGCCGGACAGCACCGGTGGTGATCTGTATATTTGTTTCAACGCAAACGAAG
AAAGCGCTAGCGCAACCCTGCCAGCTATTGCTGAAGGTAGCATGTGGCTGCGTCTGGTTGATACCAGCCTGGCGTTTCCGGGTTTCTTTAGCCGTGG
TAGCAGCCATGAAACCCACCAGGTGCTGGGTTTCAGCTCCTACCAAGTGAAAGCCCATTCCTGCGTGTTGTTTGAGTCCAAGCGTGTTTTGTCCTAA
ATCCTGAGACCTGAGACGGCATGGATCC

*Bam*HI

>ISA2-S

*Nde*I

GCATCGTCTCATCGGTCTCATATGCGTGGTGGTCTGGTGCTGCGTCCGGCTGGTAGCGGTGAAGGTGTGCCGCTGGCACCAGCTGCTCCGGGTGGTG
CTCTGGTTGCAGAACTGAGCTATGACGTGGCACGTGCGCCGTTTCATGTTAGCTTCACCCTGGCTGATGCAATGGGTGCAGAAATCCGTACCCACCG
TGGTACCAGCTTTCGTGTTCCGGTGGGTGTTGGTAGAGGTTGTCCGAGCCCGCTGGGTCTGAGCCAGAGCAAAGACGGTGCAGCGAATTTTGCAGTG
TACAGCAAAATTGCGAAGGGTATGGTTCTGTGTCTGTTCGGTGGTGGTGGTGGTGATGGTCCGGCTCTGGAAATCGAACTGGACCCGTATGTTCATC
GTACCGGTGATGTGTGGCACGTTAGCATGGAAAGCGTGGAAGGTTACGCGCGTTATGGTTTTCGTAGCGGTCTGTTTGCTATGTTCGGTATTGATCG
TCCGCTGCTGGACCCGTACGCAAAAGTGATCGGTGATTTCGTTGCGGGTGACAGCGTGGATGAAGACGGTCTGGCTGTTCCGAGCATTCGTTGCCTG
GCGAGCCTGAAGAATGCTCCGAACTACGATTGGGGTCGTGACAAACACCCGTGTCTGCCGCTGGAAAAGCTGGTTGTGTATCGTGCGAATGTTGCTC
TGTTCACCAAAGACCGTAGCAGCCGTCTGGCAGATAACGCTGCAGGTACCTTTAGCGGTATGAGCGCGAAGGTTGAACATTTCCGTCACCTGGGTGT

```
GAATGCTGTTCTGCTGGAACCGGTTTTTCCGTTCCATCAAGTGAAAGGTCCGTACTTTCCGTATCACTTTTTCAGCCCGATGAGCCTGTATAGCAGC
GAATGCAGCAGCGTGAGCGCGATTAAAAGCATGAAGGACATGGTTAAGACCATGCATCGTAACGGTATCGAAGTGCTGCTGGAAGTTGTGTTCACCC
ACACCGCAGAAGGTGGTGCGGAATGTCAAATGATTAGCCTGCGTGGTATCGACGGTAGCAGCTACTATATTGCTGATGGTATCGCAGGTTGCAAAGC
GAGCGTGCTGAATTGTAACCATCCGGTTACCCAGAAGCTGATTCTGGATAGCCTGCGTCATTGGGTTCTGGATTTTCACGTGGACGGTTTTTGCTTC
ATCAACGCTCCGTTCCTGGTTCGTGGTCCACGTGGTGAAGGTCTGAGCCGTCCGCCGCTGCTGGAAGCTATTGCTTTTGACCCGGTTCTGAGCAAAA
CCAAGATTATCGCGGACCCGTGGAGCCCGCTGGATATCAGCAATGTGCAGTTTCCGTTCCCGCACTGGAAGCGTTGGGCTGAAATGAACACCCGTTT
TAGCATGGATGTTCGTAAATTCCTGAAGGGTGAAGCTCTGATCAGCGACCTGGCAACCCGTCTGTGTGGTAGCGGTGATCTGTTCAGCAGCCGTGCA
CCGGCGTTTAGCTTCAATTACGTTAGCCGTAACAGCGGTCTGACCCTGGTGGACCTGGTTAGCTTTAGCAGCGATGAACTGGCAAGCGAATTCAGCT
GGAATTGCGGTGAAGAAGGTCCGAGCGAAAATAACGCGGTTCTGCAAACCCGTCTGCGTCAGATTCGTAACTTTCTGTTCATTCTGTTTATCAGCCT
GGGTATCCCGGTGCTGAATATGGGTGACGAATGTGGTAACAGCGCGGCTGGTAGCACCAGCTATAAAGATCGTGGTCCGCTGAATTGGAAAGCTCTG
AAGACCGCATTCGTGAAGGAAGTTACCGGTTTTATTAGCTTCCTGAGCGCGCTGCGTAGCCGTCGTGCTGATATTTTTCAACGTTGCGAATTCCTGA
AACTGGAAAACATCCATTGGTACGGTAGCGACCTGAGCGAACCGTGCTGGGAAGATCCGACCAGCAATTTTCTGTGTCTGCACATCAACGCTGAACT
GGATGAAAAGCTGCCGGACAGCACCGGTGGTGATCTGTATATTTGTTTCAACGCAAACGAAGAAAGCGCTAGCGCAACCCTGCCAGCTATTGCTGAA
GGTAGCATGTGGCTGCGTCTGGTTGATACCAGCCTGGCGTTTCCGGGTTTCTTTAGCCGTGGTAGCAGCCATGAAACCCACCAGGTGCTGGGTTTCA
GCTCCTACCAAGTGAAAGCCCATTCCTGCGTGTTGTTTGAGTCCAAGCGTGTTTTGTCCTAAATCCTGAGACCTGAGACGGCATGGATCC
                                                                                    BamHI
```

>ISA1

```
                        NdeI
GCATCGTCTCATCGGTCTCATATGGTTGCAGAAGCGGTGCAAGCAGAAGAAGATGACGATGACGATGACGAAGAAGTGGCGGAAGAACGTTTCGCTC
TGGGTGGTGCATGTAGAGTGTTGGCTGGTATGCCGGCTCCGCTGGGTGCTACCGCACTGCGTGGTGGTGTTAATTTTGCTGTGTACAGCAGCGGTGC
TAGCGCTGCTAGCCTGTGTCTGTTTGCACCGGGTGACCTGAAGGCGGACCGTGTTACCGAAGAAGTGCCACTGGACCCGCTGCTGAACAGAACCGGT
AATGTTTGGCATGTGTTTATCCACGGTGACCAACTGCACGGTATGCTGTACGGTTATCGTTTTGATGGTGTGTTCGCTCCGGAACGTGGCCAGTACT
ATGACGTTAGCAACGTTGTGGTTGATCCGTACGCGAAAGCTGTGGTTAGCCGTGGTGAATATGGTGTTCCAGCTCCGGGTGGTAGCTGCTGGCCACA
GATGGCTGGTATGATCCCGCTGCCGTATAACAAATTCGATTGGCAAGGTGACCTGCCGCTGGGTTACCATCAGAAGGATCTGGTTATCTACGAAATG
CATCTGCGTGGTTTTACCAAACACAACAGCAGCAAAACCAAGCATCCGGGTACCTACATTGGTGCGGTGAGCAAACTGGATCACCTGAAGGAACTGG
GTGTTAACTGCATCGAACTGATGCCGTGTCATGAATTCAATGAACTGGAATACTTCAGCAGCAGCAGCAAGATGAACTTCTGGGGTTATAGCACCAT
TAATTTCTTTAGCCCAATGGCGCGTTACAGCAGCAGCGGTATTCGTGATAGCGGTTGTGGTGCTATCAATGAATTCAAAGCATTCGTGCGTGAAGCG
CACAAGCGTGGTATTGAAGTTATCATGGACGTGGTTTTCAACCATACCGCAGAGGGTAATGAAAAGGGTCCGATTCTGAGCTTTCGTGGTATCGATA
ACAGCACCTACTATATGCTGGCGCCGAAGGGTGAATTTTACAACTATAGCGGTTGCGGTAATACCTTCAACTGTAATCACCCGGTGGTTCGTGAATT
CATTGTTGACTGCCTGCGTTATTGGGTGACCGAAATGCATGTTGACGGTTTTCGTTTCGATCTGGCTAGCATCCTGACCCGTGGTTGTAGCCTGTGG
GACCCGGTTAATGTGTACGGTAGCCCGATGGAAGGTGACATGATTACCACCGGTACCCCGCTGGTTGCGCCGCCGCTGATTGACATGATCAGCAACG
ATCCGATTCTGGGTAATGTTAAACTGATCGCAGAAGCGTGGGATGCAGGTGGTCTGTATCAAGTGGGCCAGTTCCCGCACTGGAACGTTTGGAGCGA
ATGGAATGGTAAATACCGTGACACCGTTCGTCAATTCATTAAGGGTACCGATGGTTTTGCTGGTGCATTCGCGGAATGCCTGTGTGGTAGCCCGCAA
CTGTATCAGGCGGGTGGTCGTAAGCCGTGGCACAGCATTAACTTTGTGTGCGCTCATGACGGTTTCACCCTGGCAGATCTGGTTACCTACAACAGCA
AATATAACCTGAGCAATGGTGAAGATAATCGTGACGGTGAAAACCACAATCTGAGCTGGAACTGTGGTGAAGAAGGTGAATTCGCGAGCCTGAGCGT
TCGTCGTCTGCGTAAGCGTCAAATGCGTAATTTCTTTGTTTGTCTGATGGTGAGCCAGGGTGTTCCGATGTTCTACATGGGTGACGAATATGGTCAT
ACCAAAGGTGGTAACAACAACACCTACTGTCATGATCACTACGTTAACTACTTCCGTTGGGACAAAAAGGAAGAACAAAGCAGCGATCTGTACCGTT
TTTGCCGTCTGATGACCAAATTCCGTAAGGAATGTGAAAGCCTGGGTCTGGAAGATTTCCCGACCAGCGAACGTCTGAAATGGCATGGTCACCAGCC
GGGTAAACCGGACTGGAGCGAAGCTAGCCGTTTTGTGGCATTCACCATGAAAGATGAAACCAAGGGTGAAATCTACGTTGCGTTTAATACCAGCCAC
CTGCCGGTGGTTGTGGGTCTGCCAGAACGTAGCGGTTTCCGTTGGGAACCGGTTGTGGACACCGGTAAAGAAGCTCCGTATGATTTTCTGACCGACG
GTCTGCCGGATCGTGCAGTTACCGTGTACCAGTTTAGCCATTTCCTGAACAGCAATCTGTACCCGATGCTGAGCTATAGCAGCATTATCCTGGTGCT
GCGTCCGGATGTTTAAATCCTGAGACCTGAGACGGCATGGATCC
                                     BamHI
```

Supplementary Figure S2. Synthetic gene sequences codon optimized for expression in yeast. Black text indicates coding regions, red text indicates linker sequences utilized for Golden Gate assembly into synthetic transcription units or classical cloning into GAL4 fusion vectors, and blue text indicates the start- and stop codons. *Bsa*I recognition sites utilized for Golden Gate assembly are double underlined. *Nde*I and *Bam*HI recognition sites used for cloning into GAL4 fusion vectors are indicated. The gene models from which these coding sequences are derived are specified in Supplementary Table S1.

A

ISA1/ISA1 and ISA1/ISA2

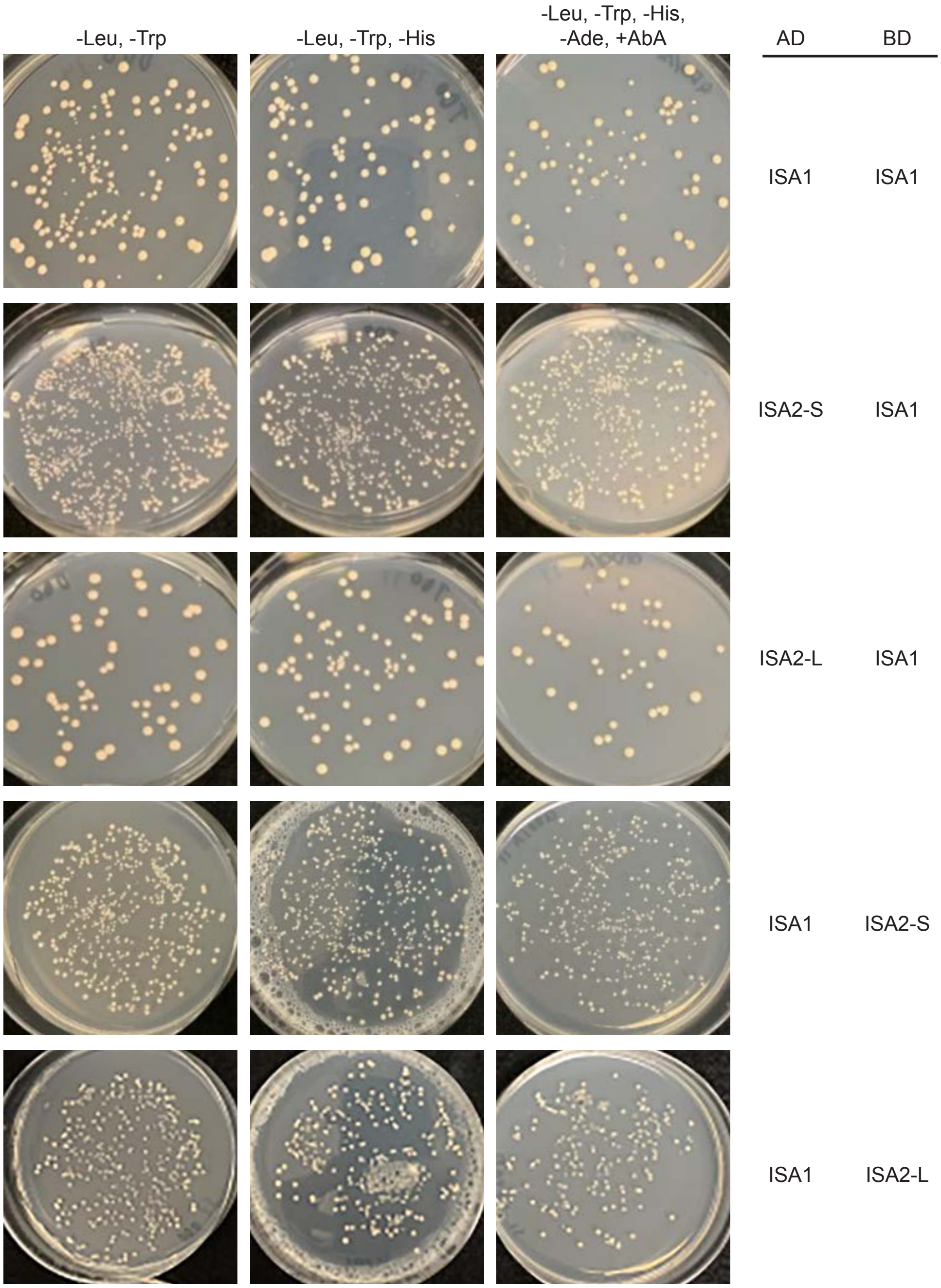

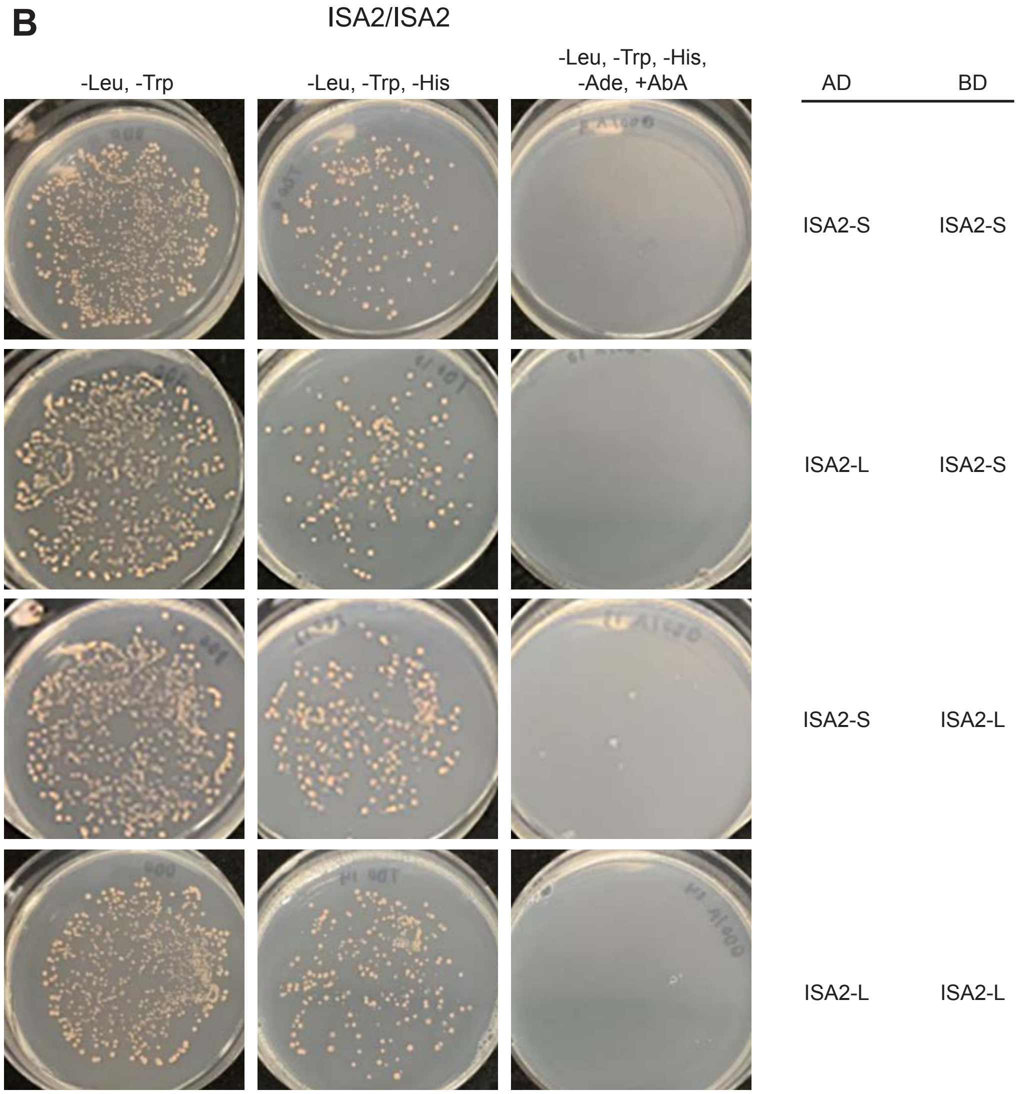
B
ISA2/ISA2
-Leu, -Trp
-Leu, -Trp, -His
-Leu, -Trp, -His, -Ade, +AbA
AD
BD
ISA2-S
ISA2-S
ISA2-L
ISA2-S
ISA2-S
ISA2-L
ISA2-L
ISA2-L

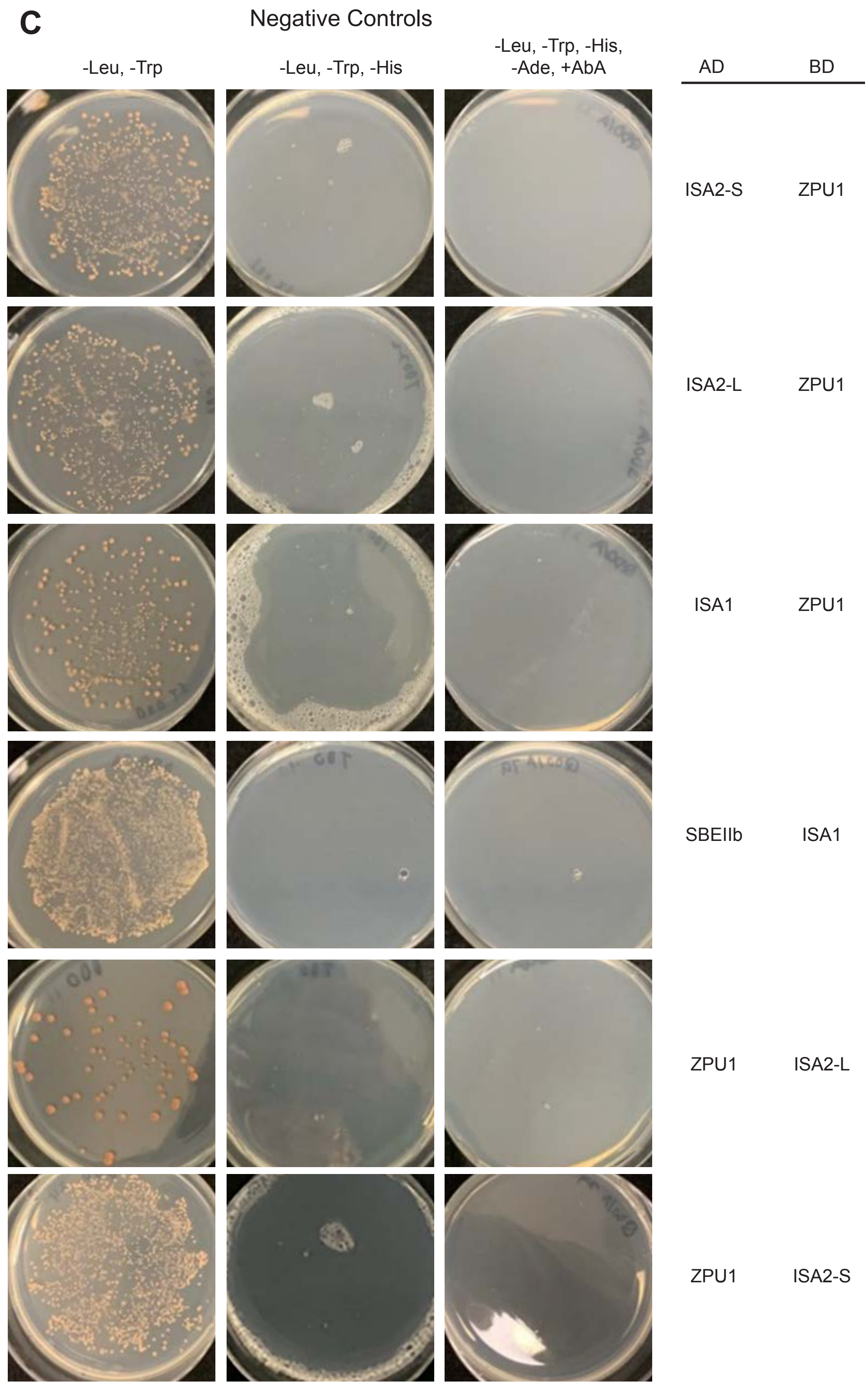

Supplementary Figure S3. *In vivo* protein-protein interaction tests accompanying Figure 3. The -Leu, -Trp plate selects for diploids created by mating of haploids expressing GAL4 activation domain (AD) fusions to partners expressing GAL4 binding domain (BD) fusions. Approximately equal numbers of diploid cells were separately deposited on relaxed test media (-Leu, -Trp, -His) or stringent test media (-Leu, -Trp, -His, -Ade, +Aureobasidin A [AbA]). Cells were grown for 2 d at 30°C prior to photographing, **A)** Interactions among ISA1 and ISA2. **B)** Interaction of ISA2 with itself. **C)** Negative results indicating that none of the fusion proteins by itself activates the histidine prototrophy marker alone, nor the three selective markers simultaneously. The last two rows are the same as the last two rows of Figure 3.

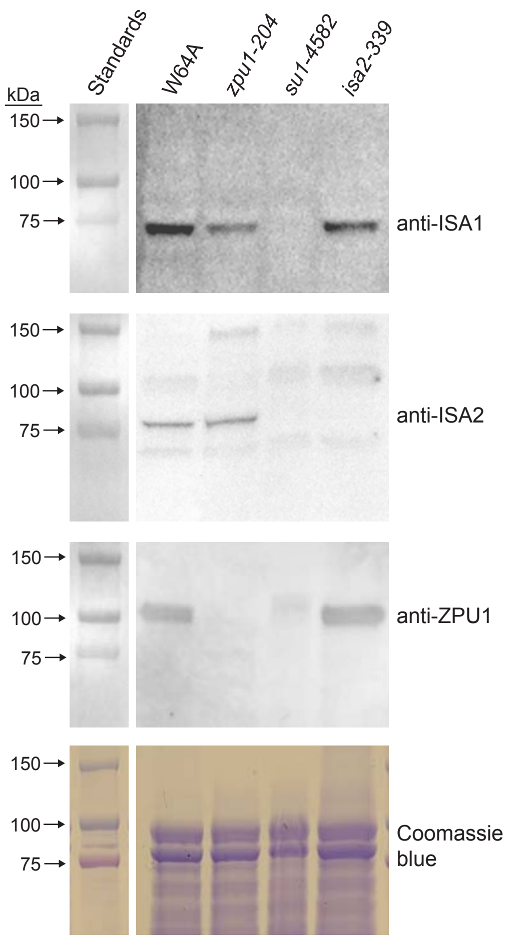

Supplementary Figure S4. Antibody specificity. The abnormal mobility of ZPU1 in the *su1-4582* mutant is shown with better resolution in Figure 2. Alleles used as controls are specified in Table 1.

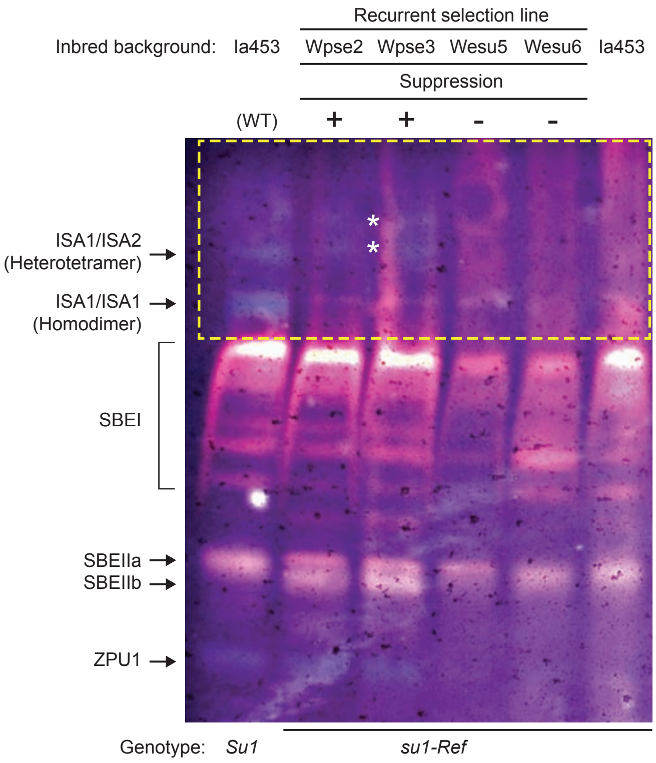

Supplementary Figure S5. In-gel enzyme activity assay of starch hydrolytic activities in recurrent selection inbreds. The portion of the zymogram in the yellow dotted line is shown in Figure 6A. Methods are as in Figure 6A. Enzyme activities responsible for each colored band were identified previously by isoform-specific antibodies, biochemical fractionation, and genetic analysis of null mutations (Kubo et al., 2010; Dinges et al., 2003a; Colleoni et al., 2003). White asterisks (*) indicate unidentified hydrolases.
